# Liquid-Phase Exfoliated GeSe Nanoflakes for Photoelectrochemical-Type Photodetectors and Photoelectrochemical Water Splitting

**DOI:** 10.1021/acsami.0c14201

**Published:** 2020-09-22

**Authors:** Gabriele Bianca, Marilena I. Zappia, Sebastiano Bellani, Zdeněk Sofer, Michele Serri, Leyla Najafi, Reinier Oropesa-Nuñez, Beatriz Martín-García, Tomáš Hartman, Luca Leoncino, David Sedmidubský, Vittorio Pellegrini, Gennaro Chiarello, Francesco Bonaccorso

**Affiliations:** † Graphene Labs, Istituto Italiano di Tecnologia, via Morego 30, 16163, Genova, Italy; ‡ BeDimensional Societa per azioni, via Albisola 121, 16163 Genova, Italy; § Dipartimento di Chimica e Chimica Industriale, Università degli Studi di Genova, via Dodecaneso 31, 16146 Genoa, Italy; ∥ Department of Inorganic Chemistry, University of Chemistry and Technology Prague, Technická 5, 166 28 Prague 6, Czech Republic; ⊥ Electron Microscopy Facility, Istituto Italiano di Tecnologia, via Morego 30, 16163 Genova, Italy; # Department of Physics, University of Calabria, Via P. Bucci cubo 31/C 87036 Rende, Cosenza, Italy; ∇ Department of Materials Science and Engineering, Uppsala University, Box 534, 75121 Uppsala, Sweden; ○ CIC nanoGUNE, 20018 Donostia-San Sebastian, Spain

**Keywords:** germanium selenide (GeSe), photocatalysts, water splitting, two-dimensional materials, hydrogen evolution reaction (HER), oxygen evolution reaction (OER)

## Abstract

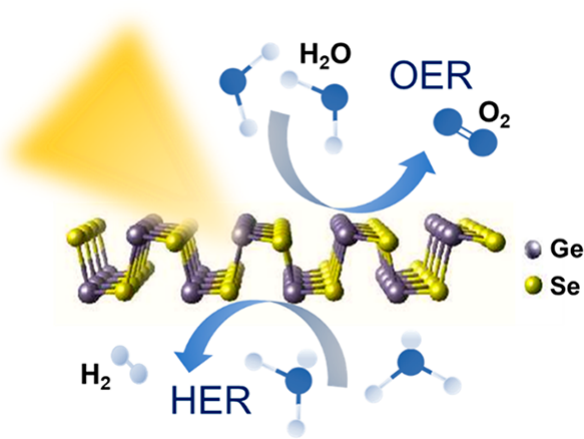

Photoelectrochemical (PEC) systems represent powerful tools to convert electromagnetic radiation into chemical fuels and electricity. In this context, two-dimensional (2D) materials are attracting enormous interest as potential advanced photo(electro)catalysts and, recently, 2D group-IVA metal monochalcogenides have been theoretically predicted to be water splitting photocatalysts. In this work, we use density functional theory calculations to theoretically investigate the photocatalytic activity of single-/few-layer GeSe nanoflakes for both the hydrogen evolution reaction (HER) and the oxygen evolution reaction (OER) in pH conditions ranging from 0 to 14. Our simulations show that GeSe nanoflakes with different thickness can be mixed in the form of nanoporous films to act as nanoscale tandem systems, in which the flakes, depending on their thickness, can operate as HER- and/or OER photocatalysts. On the basis of theoretical predictions, we report the first experimental characterization of the photo(electro)catalytic activity of single-/few-layer GeSe flakes in different aqueous media, ranging from acidic to alkaline solutions: 0.5 M H_2_SO_4_ (pH 0.3), 1 M KCl (pH 6.5), and 1 M KOH (pH 14). The films of the GeSe nanoflakes are fabricated by spray coating GeSe nanoflakes dispersion in 2-propanol obtained through liquid-phase exfoliation of synthesized orthorhombic (*Pnma*) GeSe bulk crystals. The PEC properties of the GeSe nanoflakes are used to design PEC-type photodetectors, reaching a responsivity of up to 0.32 AW^–1^ (external quantum efficiency of 86.3%) under 455 nm excitation wavelength in acidic electrolyte. The obtained performances are superior to those of several self-powered and low-voltage solution-processed photodetectors, approaching that of self-powered commercial UV–Vis photodetectors. The obtained results inspire the use of 2D GeSe in proof-of-concept water photoelectrolysis cells.

## Introduction

The conversion of light energy into chemical fuels and electricity through photoelectrochemical (PEC) cells represents a powerful strategy for sustainable fuel and chemical generation,^1−4^ environmental remediation (i.e., pollutant degradation),^5−7^ advanced analytical systems (i.e., chemical sensors) for environmental^8,9^ and biological monitoring,^9−11^ as well as innovative self-powered photodetectors.^12,13^ In particular, PEC water splitting is envisioned to produce molecular hydrogen (H_2_),^14,15^ seen as an ideal energy carrier for the storage and distribution of solar energy in the so-called “Hydrogen economy”.^16,17^ In addition, aqueous PEC cells, including water splitting ones, are emerging for the development of inexpensive, easily fabricated, environmentally friendly self-powered photodetectors with high spectral responsivity (>tens of mA W^–1^ in UV–visible spectral region),^12,18−20^ fast response (in the order of tens of ms)^12,19,21^ and satisfactory sensitivity (typically in the order of 10).^12,19,22^ To achieve efficient PEC systems, it is necessary to develop photocatalytic materials that efficiently absorb light in the desired spectral range (UV–visible for solar energy conversion systems),^23^ creating free charge carriers with suitable energies to accomplish the targeted oxidation–reduction (redox) reactions before they recombine.^23−25^

In this context, two-dimensional (2D) materials, including either single- or few-layer flake forms, are attracting ultimate interest as potential advanced photo(electro)catalysts.^26−29^ Such attention mainly relies on their large surface-to-volume ratio, which guarantees that the charge carriers, i.e., electrons and holes, are directly photogenerated at the interface with the electrolyte, in which redox reactions take place before charge recombination.^26−29^ Both theoretical and experimental works have investigated the photocatalytic water splitting properties of graphene derivatives,^30−33^ as well as other 2D materials, including graphitic carbon nitrides,^34−36^ transition metal dichalcogenides (e.g., MoS_2_,^37,38^ WS_2_,^39,40^ and ReS_2_^41,42^) transition metal oxides^43^ (e.g., H^+^/K_4_Nb_6_O_17_,^44^ TBA^+^/Ca_2_Nb_3_O_10_^45^), (functionalized) monoelemental materials (e.g., phosphorene,^46−49^ germanane,^50^ and silicene^50^), MXenes,^51^ group-IVB metal nitride halides,^52^ group-IIB metal monochalcogenides (e.g., ZnSe,^53,54^), and group-IIIA metal monochalcogenides^55−57^ (e.g., GaSe^58^ and InSe^59,60^).

Recently, 2D group-IVA metal monochalcogenides (MX, M = Si, Ge, Sn, Pb; X = S, Se, Te), namely SiS, SiSe, SiTe, GeS, GeSe, GeTe, SnS, and SnSe, have been theoretically predicted to be low-cost and environmentally friendly water splitting photocatalysts.^61−68^ However, the field evaluation of their photo(electro)catalytic properties is still missing, pointing out the need for experimental trials and validation. Theoretical studies revealed that their monolayer form is stable both in the phosphorene-derived distorted NaCl-type structure (“black-phase structure”, space group: *Pmn*2_1_),^69−76^ and the *Pma2* structure.^69,77^ However, a large variety of polymorphisms, including blue-phosphorene-like *Cmcm* structure,^72,75,76,78−82^ cubic polymorph,^83^*Fm*3̅*m* structure,^84,85^*P*2_1_*ca* structure,^81,86−88^ and *P*4/*nmm*,^70^ have been synthesized at high temperature or pressure,^83,89^ and/or predicted to be metastable.^70,87,88^ Importantly, each material polymorph shows distinctive optoelectronic properties,^90^ which can be further tuned by strain engineering,^76,91−94^ thus creating a material platform for novel nanoelectronics. Among the plethora of 2D group-IV metal monochalcogenides, GeSe polymorphs have been deeply investigated for application in several fields, including photovoltaics,^95−98^ photodetectors,^82,99−105^ (tunnel) field-effect transistors,^106−109^ spintronic,^110,111^ piezoelectric actuators,^88,112^ and ferroelectric devices,^113^ and energy storage systems,^114−117^ beyond to be proposed as water splitting photo(electro)catalysts.^61,62,68,93^ Density functional theory (DFT) calculations revealed that its cleavage energy from the corresponding orthorhombic bulk structure is around 0.45 J m^–2^,^62^ which is similar or slightly superior to those calculated for other 2D materials, including graphene (0.3–0.4 J m^–2^,^118,119^ experimental value: 0.37 J m^–2^),^120^ several transition metal dichalcogenides^121^ (e.g., MoS_2_, 0.29 J m^–2^),^121^ group-V elemental materials (e.g., phosphorene, 03–0.4 J m^–2^),^122,123^ and several group-IIIA metal monochalcogenides (e.g., GaSe, 0.29 J m^–2^).^57,58^ These results suggest that 2D GeSe can be easily produced through the exfoliation of its bulk counterpart, including either micromechanical cleavage-based exfoliation^124−126^ or scalable liquid-phase exfoliation (LPE) methods.^124,127−129^ The exfoliation of GeSe crystal has been experimentally established for both fundamental and applied research.^68,101−104,107−109,130,131^ In comparison to its phosphorene analogues, the “black-phase” 2D GeSe structure shows a superior oxidation resistance,^68,132,133^ with activation energies for the chemisorption of O_2_ on its surface of 1.44 eV (more than twice of the value calculated for phosphorene).^133^ Moreover, theoretical studies reported that the presence of H_2_O molecules does not influence the oxidation process of Ge-based monochalcogenides,^133^ which is different from the cases of isostructural phosphorene^133−135^ and group-IIIA metal monochalcogenides (e.g., InSe^136−138^ and GeSe^139,140^). Therefore, these results advise a feasible use of 2D GeSe into photo(electro)chemical devices. To this end, the optoelectronic properties of the exfoliated GeSe can be tuned by varying the number of layers.^62,68^ In fact, theoretical calculations demonstrated a *c*-axis confinement-induced optical bandgap (*E*_g_) blue-shift,^62,68^ showing an indirect bandgap in the bulk (between 1.1 and 1.2 eV)^141−143^ and a direct bandgap in the monolayer (>1.9 eV).^62,68^ This *E*_g_ evolution from bulk to monolayer resembles the one exhibited by several group-VI transition metal dichalcogenides^144^ (e.g., MoS_2_^145−147^ and MoSe_2_^148^). Additionally, the *E*_g_ of single-/few-layer GeSe flakes is larger than the minimum energy required for the water splitting reaction (i.e., 1.23 eV).^23^ Even more, the number of layers in 2D GeSe determines the energy of conduction band minimum (CBM) and valence band maximum (VBM), which can be adjusted to fulfill the fundamental requirements for a water splitting photo(electro)catalysts, i.e.: 1) CBM energy (*E*_CBM_) > reduction potential of H^+^/H_2_ (*E*(*H*^*+*^/*H*_*2*_)), 2) VBM energy (*E*_VBM_) < reduction potential of O_2_/H_2_O (*E*(O_2_/H_2_O)).^61,62,68^ The interest for 2D GeSe as a photo(electro)catalyst also arises from its unusually strong visible-light absorbance (absorption coefficient up to 10^5^ cm^–1^ in the visible spectral range).^149^ The latter has been ascribed to the multiple electronic bands that are displayed near both VBM and CBM.^62,149^ These electronic bands originate from the large joint density of states, which gives rise to a large probability of optical transitions across the energy gap.^97,143^ Moreover, the electronic structure of GeSe results in low excitonic binding energy, predicted to be even lower than 100 meV,^62,97^ and indicating efficient excitons dissociation in free charges.^62,97^ Even more, the GeSe charge carriers have high charge carrier mobility (theoretical values between 10^2^ and 10^4^ cm^2^ V^–1^ s^–1^ for electrons,^62,150−152^ between 1 and 10^3^ cm^2^ V^–1^ s^–1^ for holes^62,104,150,151,153^), facilitating their migration to the material surface in which the redox processes take place.^62^

Stimulated by the predicted properties of 2D GeSe, we report the first experimental demonstration of the photo(electro)catalytic activity of single-/few-layer GeSe flakes in different aqueous media, ranging from acidic to alkaline solutions (i.e.: 0.5 M H_2_SO_4_, pH 0.3; 1 M KCl, pH 6.5; 1 M KOH, pH 14). Theoretical calculations were used to evaluate the electronic structures of single- and few-layer GeSe flakes and bulk GeSe. We describe the PEC working mechanisms of the photoelectrode based on GeSe nanoflakes with heterogeneous morphological properties such as lateral size and thickness, resulting in different (opto)electronic and photocatalytic properties. We reveal that GeSe nanoflakes with different thicknesses in nanoporous electrodes can act as different light absorbers in nanoscale tandem systems, mimicking photosynthetic systems (similarly to bioinspired molecular photocatalysts),^154,155^ by creating monolithic “all-solid-state Z-scheme water splitting pathways”.^156−159^ These expectations are experimentally proven on photoelectrodes fabricated through the spray-coating of single- and few-layer GeSe flakes, which are produced through the LPE of a synthetized “black-phase” GeSe crystal in an environmentally friendly solvent (i.e., isopropyl alcohol, IPA). The electrochemical characterization of our GeSe-based photoelectrodes proves both photoanodic and photocathodic responses in aqueous media, allowing PEC-type photodetectors for visible light to be conceived. Next, GeSe photoelectrodes are characterized after simulated sunlight for water splitting reactions, hydrogen evolution reactions (HER), and oxygen evolution reactions (OER).

## Results and Discussion

### Understanding of Structural, Optoelectronic, and Catalytic Properties of the GeSe Nanoflakes

**Figure 1:**
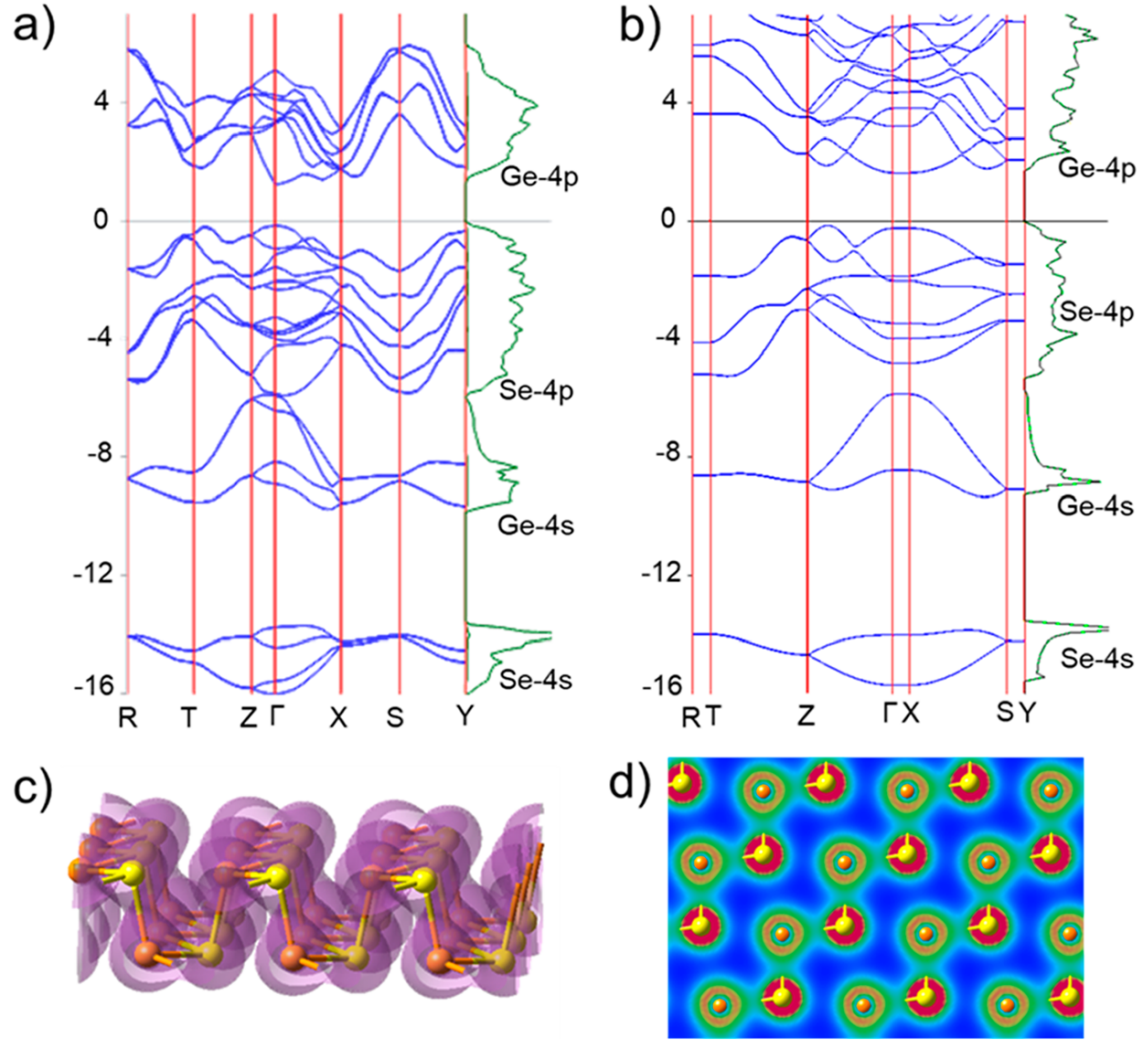
(a,b) Band dispersion along principal directions of the first Brillouin zone and density of states for B-GeSe and 1L-GeSe, respectively. (c) 3D isosurface of the electron density = −0.3 *e* Å^–3^. (d) Electron density distribution in 2D cross section over one Ge–Se layer.

The thermodynamic requirements for a water splitting photocatalyst are *E*_VBM_ < *E*(O_2_/H_2_O) and *E*_CBM_ > *E*(H^+^/H_2_) for the OER and the HER, respectively.^23,160^ Therefore, the electronic structure calculation by means of DFT with generalized gradient approximation (GGA-PBE96)^161^ and Heyd-Scuseria-Ernzerhof hybrid exchange-correlation functional (HSE06)^162^ for bulk GeSe (B-GeSe) and single-/few-layer GeSe (*x*L-GeSe, *x* = 1; 2; 4 and 6) (see details in the Experimental Section of the Supporting Information, SI) were carried out to verify that GeSe nanoflakes fulfill the energetic requirements for PEC water splitting. A clear feature of the ground state polymorph of B-GeSe (space group *Pnma*) is that it is not only isostructural but also isoelectronic with black phosphorus.^68,71^ Therefore, as shown in Figure 1a, B-GeSe reveals noticeable similarities to the parent structure of black phosphorus.^68^ Nevertheless, compared to black phosphorus, the difference in electronegativities and hence in on-site energies of Ge- and Se-4*s* and 4*p* valence orbitals imposes a larger and indirect bandgap (1.35 eV), as well as a larger energy separation between the Se-4*s* band (centered at 14 eV below the Fermi level, *E*_F_) and the topmost valence band of predominant Se 4*p* character (spreading from *E*_F_ down to −6 eV).^97,143^ Near the bottom of this valence band there is a Ge-4*s* band arising from Ge(2+)-4*s*^*2*^ electron configuration. The comparison of the band dispersion for B-GeSe (Figure 1a) and 1L-GeSe (Figure 1b) shows a bandgap broadening with a decreasing number of layers (from 1.35 eV in B-GeSe to 1.80 eV in 1L-GeSe). In both cases, the bandwidth of ∼3 eV and its hybridization with Se-4*p* states indicate that Ge-4*s*^*2*^ pair is far from being nonbonding and is less stereoactive than P-3*s*^*2*^ in black phosphorus or phosphorene.^163,164^

These attributes are further supported by the plots of electron density (Figure 1c,d), revealing a nearly spherical distribution around Ge atoms (the red spots in panel d correspond to semicore Ge-3*d* states). By contrast, the Ge-4*p* orbitals are unoccupied and give rise to three different anisotropic conduction bands per Ge atom in the unit cell. The resulting spatial separation of the photogenerated carriers (i.e., holes on Se and electrons on Ge) could be effective to suppress the electron–hole recombination in GeSe, promoting efficient photo(electro)catalytic responses.^165^ The work function (WF) evolution of *x*L-GeSe with the number of layers was also elucidated through DFT calculations, showing WF values from 2.2 eV for 1L-GeSe to 5.3 eV for 6L-GeSe, and the upper limit value of 6.5 eV for B-GeSe. The values of the bandgap were combined with the calculated WFs to construct the plot of *E*_VBM_ and *E*_CBM_ vs the vacuum level, aiming to evaluate the *E*_VBM_ and *E*_CBM_ of xL-GeSe and B-GeSe relative to *E*(O_2_/H_2_O) and *E*(H^+^/H_2_), respectively.

**Figure 2:**
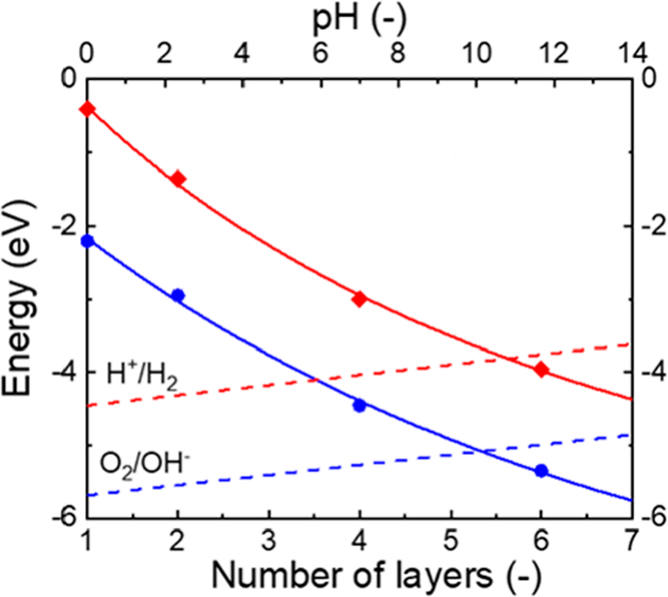
*E*_VBM_ (lower solid blue curve and ● symbols) and *E*_CBM_ (upper solid red curve and ⧫ symbols) of GeSe as a function of its layer number, compared with the potentials of water splitting (*E*(H^+^/H_2_) and *E*(O_2_/H_2_O)) as a function of pH.

Figure 2 reports the *E*_CBM_ and the *E*_VBM_ of *x*L-GeSe as functions of the number of layers, while showing the *E*(*H*^*+*^/H_2_) and *E*(O_2_/H_2_O) as functions of the pH. The theoretical pH window satisfies the condition *E*_CBM_ > *E*(H^+^/H_2_) and *E*_VBM_ < *E*(O_2_/H_2_O) for the HER and the OER, respectively, corresponding to the pH range ∼10.2–10.8 and number of layers ∼5–6. However, *x*L-GeSe with *x* < 6 fulfill the HER photocatalyst requirement independently of the pH. Vice versa, *x*L-GeSe with *x* ≥ 6 can satisfy the OER photocatalyst requirement. Therefore, GeSe nanoflakes with different thickness can be interfaced to act as nanoscale tandem systems, in which the thinner nanoflakes (e.g., *x*-GeSe with *x* ≤ 4) preferably operate as HER-photocatalysts, while the thicker ones (e.g., *x*-GeSe with *x* > 4) can catalyze the OER (depending on the pH of the medium). By creating monolithic “all-solid-state Z-scheme water splitting pathways”,^156−159^ such GeSe systems could mimic the working processes of photosynthetic structures.^154,155^ The van der Waals (vdW) interactions represent an essential feature in the modeling of GeSe, since they held together the different layers in the bulk stacks.^166,167^ Our DFT calculations using GGA-PBE96 and including the vdW dispersion correction through the DFT-D3 method reveal more negative energy (by 25.5 kJ mol^–1^) of B-GeSe compared to 1L-GeSe. Moreover, the surface energies obtained from the slab structures calculations decrease from 250 mJ m^–2^ for 1L-GeSe to 226 mJ m^–2^ for 6L-GeSe. From the polynomial curve fitting the data, the surface energy can be estimated to be ∼220 mJ m^–2^ for the B-GeSe (001) surface. This value is comparable to other p-block chalcogenides, such as GaSe (145 mJ m^–2^)^58^ and graphene (185 mJ m^–2^).^120^ Being the cleavage energy the surface energy, our theoretical data support that *x*L-GeSe can be produced by cleaving the B-GeSe, similar to the exfoliation of other type of layered materials.^120^

### Synthesis of Exfoliation GeSe Crystals and Material Characterization

**Figure 3:**
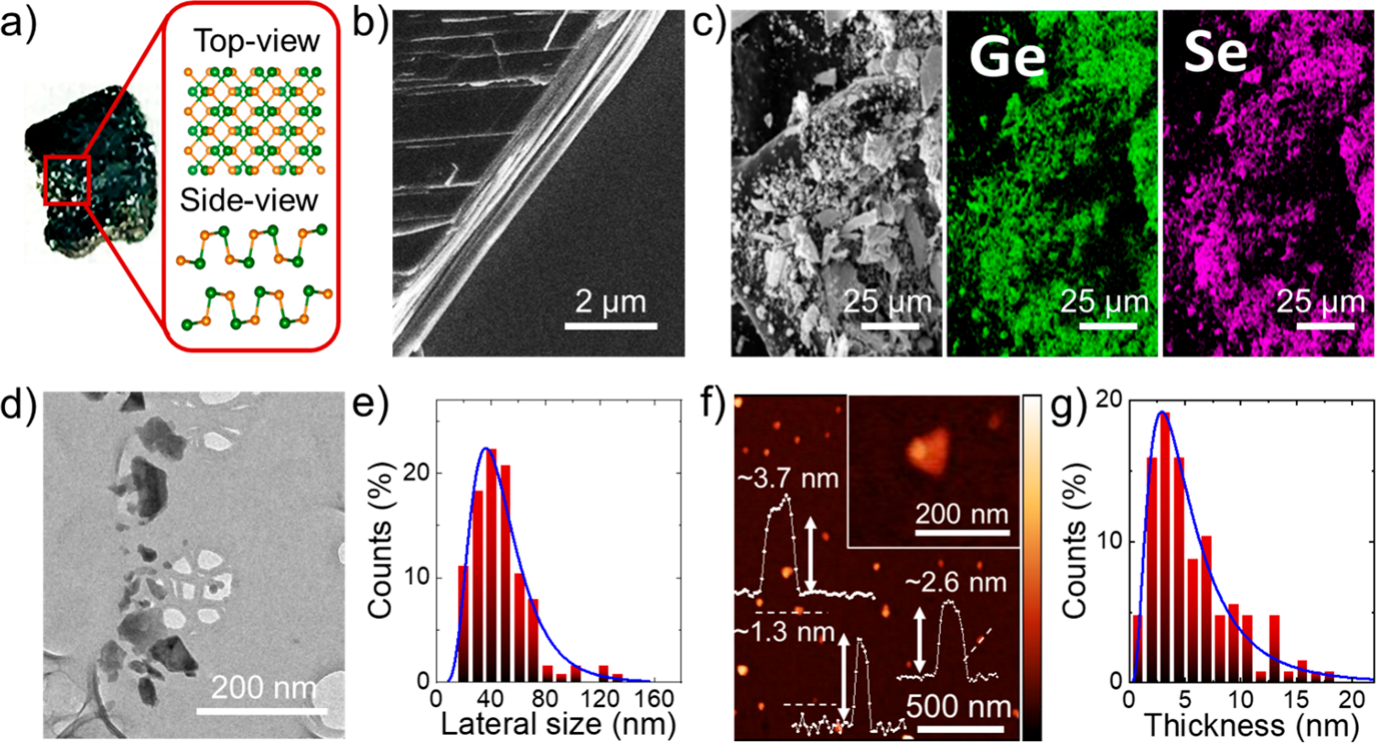
(a) Photograph of a GeSe crystal synthesized through the controlled cooling method. The orthorhombic (*Pnma*) structure of the GeSe crystal is also shown. (b) SEM image of a fragment of GeSe crystal, evidencing its layered structure. (c) SEM image of fragments of GeSe crystals and the corresponding EDS maps for Ge (*K*α = 9.9 eV, green) and Se (*L*α = 1.4 eV, violet). (d) BF-TEM image of the GeSe nanoflakes produced through LPE of pulverized GeSe crystal. (e) TEM statistical analysis of the lateral dimension of representative GeSe nanoflakes. (f) AFM image of representative GeSe nanoflakes. Height scale bar: 10 nm. The height profiles of two sections are also shown, exhibiting the presence of single-/few-layer flakes. (g) AFM statistical analysis of the thickness of the GeSe nanoflakes.

Orthorhombic (*Pnma*) GeSe crystals were produced through direct synthesis followed by slow cooling of melt granules of Ge and Se elements.^168^ Briefly, powders of Ge and Se with an elemental stoichiometry of 1:1 were inserted in a quartz glass ampule, and afterward evacuated, sealed, and heated at 800 °C (i.e., above melting temperature of GeSe) for 1 h (heating rate = 5 °C min^–1^). The obtained products were cooled down to room temperature (cooling rate = 0.3 °C min^–1^), obtaining the GeSe crystals. Figure 3a shows a photograph of a representative GeSe crystal, together with its crystal structure consisting of double-layer slabs of Ge–Se in a zigzag configuration, separated from one another by a van der Waals gap.^91,169^ The morphology of the GeSe crystals was evaluated by scanning electron microscopy (SEM) coupled with energy-dispersive X-ray spectroscopy (EDS). The high-magnification SEM image of an edge of a fragment of the GeSe crystal (Figure 3b) evidences its layered structure. The SEM/EDS analysis (Figure 3c and Table S1) shows a slight excess of Ge (Ge-enriched phases) of the GeSe crystals (Ge-to-Se atomic ratio ∼1.2). The stoichiometric excess of Ge is associated with the presence of corresponding oxides (i.e., GeO_2_ and GeO), which could form from the oxidation of Ge reactant residuals during air exposure.^170,171^

The GeSe nanoflakes were obtained through the LPE of pulverized GeSe crystals in anhydrous IPA. Importantly, IPA has been previously used to successfully exfoliate other Ge-based monochalcogenides (i.e., GeS)^172^ or group-IIIA metal monochalcogenides (e.g., GaSe,^58^ GaS,^173^ and InSe^174,175^). Moreover, it circumvents the processability issues related to the use of high-boiling point and toxic solvents often used for the exfoliation of layered materials,^176,177^ e.g., *N*-Methyl-2-Pyrrolidone (NMP) for graphite^178,179^ and several metal chalcogenides.^180−182^ Subsequently, the dispersion was centrifuged to separate the unexfoliated pieces of crystals (sediment) from the GeSe nanoflakes (a process known as sedimentation-based separation, SBS),^183−185^ which were collected by extracting 80% of the supernatant. Transmission electron microscopy (TEM) and atomic force microscopy (AFM) analyses were performed to investigate the morphology of the GeSe nanoflakes. Figure 3d shows a Bright Field TEM (BF-TEM) of the GeSe nanoflakes, which display irregular shapes with sharp edges. The statistical analysis of their lateral sizes (Figure 3e) shows values ranging from 15 to 180 nm and following a log-normal distribution peaked at ∼36 nm.

**Figure 4:**
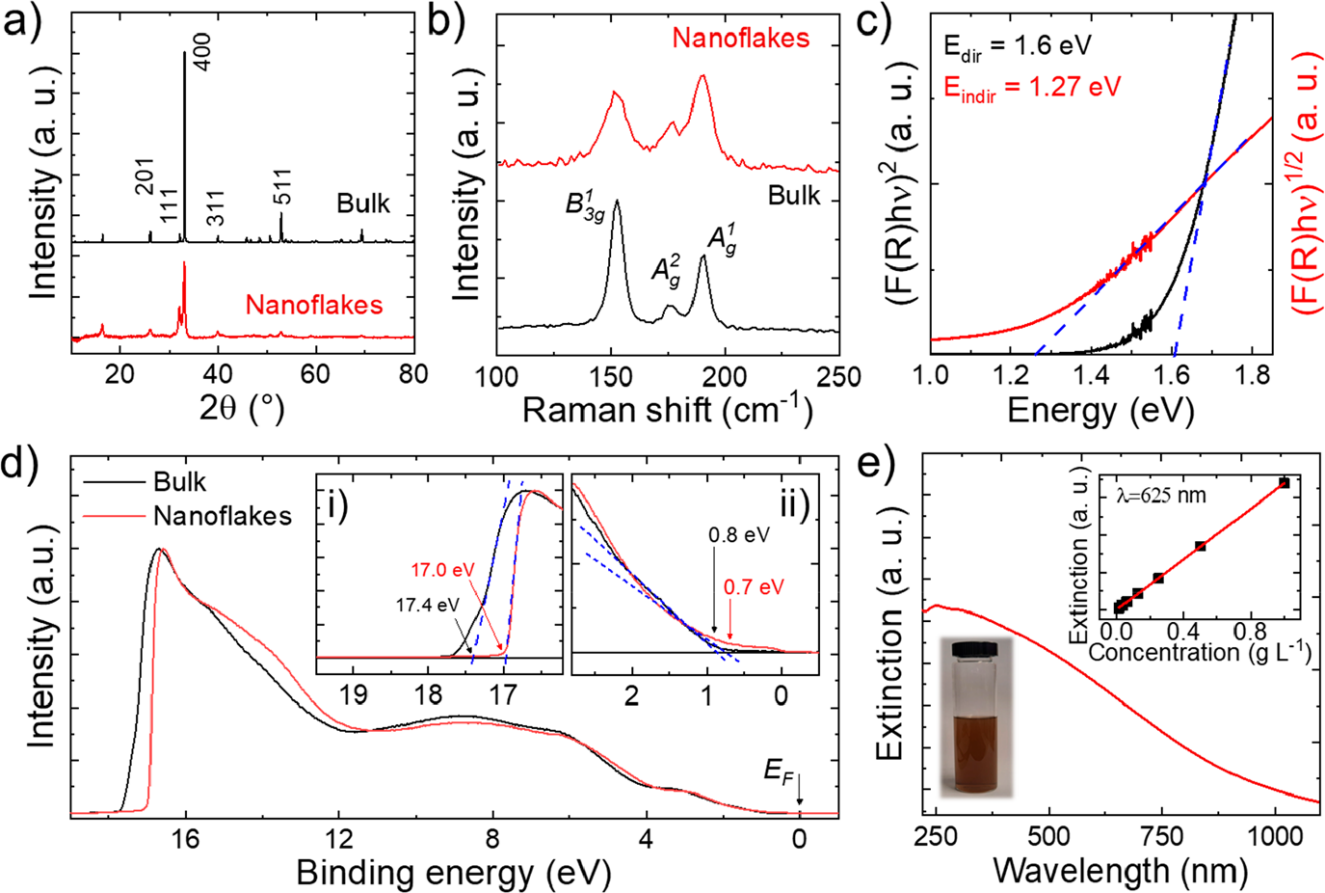
(a) XRD diffractograms and (b) Raman spectra (excitation wavelength = 633 nm) of GeSe bulk (fragments of the as-synthesized GeSe crystal) and nanoflakes. The panels respectively report the diffraction peaks and the Raman modes attributed to the orthorhombic structure of the GeSe. (c) (*F*(*R*)*h*ν)^*n*^ vs *h*ν (Tauc plots) for the GeSe nanoflakes for both direct (*n* = 2) and indirect interband transitions (*n* = 0.5). (d) UPS spectra for GeSe bulk (fragments of the as-synthesized GeSe crystal) and nanoflakes (the binding energy is relative to the *E*_F_, i.e., *E*_F_ = 0 eV). The inset panel (i) shows the enlargement of the secondary electron cutoff region of the spectra, while the inset panel (ii) reports the region near the *E*_F_ (i.e., VBM region) of the spectra. (e) Optical extinction spectrum Ext(λ) of the LPE-produced GeSe nanoflake dispersion, the photograph of which is also shown in the panel. The top-right inset panel reports the Ext(625 nm) vs *c* plot.

Figure 3f reports an AFM image of various nanoflakes, with their height profiles (values between 1.1 and 7.5 nm). The statistical analysis of the thickness of the nanoflakes (Figure 3g) indicates that the values follow a log-normal distribution peaked at 2.8 nm. By considering an (experimental) AFM thickness of monolayer GeSe between 1 and 1.5 nm,^186^ close to calculated values,^91,187^ our AFM data indicates that the exfoliated sample is predominantly made of few (≤5)-layer flakes. However, either single layer or multi (>5)-layer flakes are present, giving a mixture of nanoflakes with different optoelectronic properties (as predicted by the DFT calculations discussed above). X-ray diffraction (XRD) patterns of GeSe bulk and nanoflakes (Figure 4a) confirm the orthorhombic (*Pnma*) structure of bulk GeSe with the following lattice parameters: *a* = 10.8200 Å, *b* = 3.8520 Å, *c* = 4.4030 Å (ICDD card Nr. 33–582). Since no extra peaks attributed to oxides appear in the XRD pattern of exfoliated samples, we conclude that the LPE in anhydrous IPA effectively preserves the native structural properties of the GeSe bulk counterpart. The *Pnma* structure of the exfoliated sample was also assessed by selected-area electron diffraction (SAED) analysis of the TEM image of GeSe nanoflakes (Figure S1). The structural properties of the GeSe bulk and nanoflakes were further evaluated through Raman spectroscopy. The group theory predicts 12 Raman active optical modes for the *D*_2*h*_^16^ space group of orthorhombic (*Pnma*) GeSe, i.e.: 4*A*_*g*_ + 2*B*_1*g*_ + 4*B*_2*g*_ + 2*B*_3*g*_.^188−190^Figure 4b shows the Raman spectra (excitation wavelength – λ_exc_– = 633 nm) of the bulk and the exfoliated GeSe samples, focusing on the spectral range of the most intense Raman peaks at ∼152, ∼176, and ∼190 cm^–1^. These peaks are respectively attributed to the out-of-plane vibration mode *B*_3*g*_^1^ and two in-plane vibration modes *A*_*g*_^2^ and *A*_*g*_^1^,^188−190^ as reported in previous studies using the same λ_exc_^191,192^ (*A*_*g*_^2^ is often not discussed in literature since it is almost negligible for λ_exc_ = 532 nm^190,191^). The comparison of our Raman spectra indicates that the ratio between the intensity of *B*_3*g*_^1^ and *A*_*g*_^1^ decreases with decreasing the thickness of the GeSe nanoflakes, in agreement with previous studies.^68,186^ In addition, *A*_*g*_^2^ is slightly blue-shifted with decreasing the thickness of the GeSe crystals, while *B*_3*g*_^1^ and *A*_*g*_^1^ approximately retain their peak positions (see quantitative Raman analysis in Figure S2). Although isostructural analogues of GeSe (e.g., black phosphorus) can exhibit reproducible thickness-dependent shifts of their Raman peaks as a consequence of the variation of the interlayer forces when the number of layer changes,^193,194^ discordant results have been reported for GeSe.^68,186^ Therefore, contrary to graphene and many 2D crystals and hybrid nanostructures,^195−197^ caution is still needed when Raman spectroscopy is used as a tool for the precise determination of the thickness of exfoliated GeSe. More importantly for our purposes, GeSe nanoflakes do not exhibit any peaks attributed to Raman active modes of other species beyond GeSe (e.g., GeO_2_ or crystalline Se modes at ∼420^198,199^ and ∼240 cm^–1^,^200^ respectively), further supporting that the LPE in IPA does not cause any relevant oxidation effects, in agreement with XRD (Figure 4a) and X-ray photoelectron spectroscopy (XPS) analyses (Figures S3 and S4, respectively). The *E*_g_ of the GeSe nanoflakes was assessed through diffusive reflectance spectroscopy (DRS) using the Kubelka–Munk theory of the diffusive reflectance (*R*).^201,202^

In particular, the *E*_g_ can be estimated by fitting the linear part of (*F*(*R*)*h*ν)^*n*^ vs *h*ν (Tauc Plot) with (*F*(*R*)*h*ν)^*n*^=*Y*(*h*ν – *E*_g_) (Tauc relation), in which *F*(*R*) is the Kubelka–Munk function, defined as *F*(*R*) = (1 – *R*)^2^/2*R*, *h* is Planck’s constant, ν is the photon’s frequency, and *Y* is a proportionality constant.^201,202^ The value of *n* specifies the type of the electronic transitions, distinguishing between direct (*n* = 2) and indirect interband transitions (*n* = 0.5).^203−205^Figure S5 reports the *R* spectrum of a film of GeSe nanoflakes deposited on quartz substrate. Figure 4c shows the corresponding Tauc plots for both *n* = 2 and *n* = 0.5, from which we estimated an indirect *E*_g_ of 1.27 eV and a direct *E*_g_ of 1.60 eV, respectively. These *E*_g_ values agree with those calculated through DFT simulations and resemble those previously reported for few-layer GeSe flakes.^68,186^ Noteworthily, our films are made of GeSe nanoflakes with polydisperse morphological characteristics, which means that the optical features of the thickest nanoflakes could experimentally screen those of thinnest nanoflakes, which shows the highest *E*_g_.^58,206^ The WF and the *E*_VBM_ of the bulk and exfoliated GeSe samples were determined through ultraviolet photoelectron spectroscopy (UPS).^203^Figure 4d reports the He I (21.22 eV) UPS spectra measured for the GeSe bulk and nanoflakes. The secondary electron cutoff region of the spectra (inset panel (i)) shows that the cutoff energies are ∼17.4 eV for GeSe bulk and ∼17.0 eV for GeSe nanoflakes, corresponding to a WF of 3.8 eV for GeSe bulk and 4.2 eV for GeSe nanoflakes. The region near the *E*_*F*_ (i.e., VBM region) of the UPS spectra (inset panel (ii)) reveals that *E*_VBM_ is −4.6 eV for the GeSe bulk and −5.0 eV for the GeSe nanoflakes. Contrary to our DFT calculations, these results indicate that the VBM of the nanoflakes is deeper than that of the bulk. Noteworthily, the WF and band gap values for surface states can be substantially affected by the chemical modification of the surface due to interaction with the surroundings, thus explaining discrepancies between experimental and theoretical data. The measured *E*_CBM_ of the GeSe nanoflakes, calculated by assuming the *E*_*g*_ previously estimated by the Tauc analysis, is 3.7 eV, which resembles the values theoretically derived for 5L GeSe flakes. As commented above for the Tauc analysis, the electronic characteristics attributed to the thinnest nanoflakes (i.e., single-/bilayer flakes) could be experimentally inaccessible through UPS measurements of a sample with nanoflakes having different thicknesses,^58,206,207^ the thicker (and larger) nanoflakes being the main contributors to weight (or atomic) composition. The concentration of the as-produced GeSe flakes dispersion was first measured by weighing the solid material content in a known volume of the dispersion, giving a value of 0.22 ± 0.02 g L^–1^. The extinction coefficient of the GeSe nanoflakes was estimated using the Lambert–Beer law: Ext(λ) = ε(λ)*cl*, in which λ is a given optical wavelength, Ext(λ) is the optical extinction at the given λ, ε(λ) is the extinction coefficient at the given λ, *c* is the material concentration, and *l* is the optical path length.^208^ In fact, by measuring the optical extinction spectra of controlled dilutions/concentrations of the as-produced GeSe nanoflake dispersion, ε(λ) is calculated from the slope of Ext(λ) vs *c* plot, being: slope = ε(λ)*l*. Once known ε(λ), *c* can also be precisely controlled among different batches of materials, being *c* = Ext(λ)/(ε(λ)*l*).

Figure 4e shows the optical extinction spectrum of the as-produced GeSe nanoflakes, which are capable to absorb the solar radiation in broad spectral range (UV–visible and near-infrared (<1100 nm) wavelengths). The inset panel reports the Ext(625 nm) vs *c* plot, whose linear fitting provides a slope corresponding to a ε(625 nm) of 136.0 L g^–1^ m^–1^.

### Photoelectrochemical Properties of the GeSe Nanoflakes and Their PEC-Type Photodetectors

**Figure 5:**
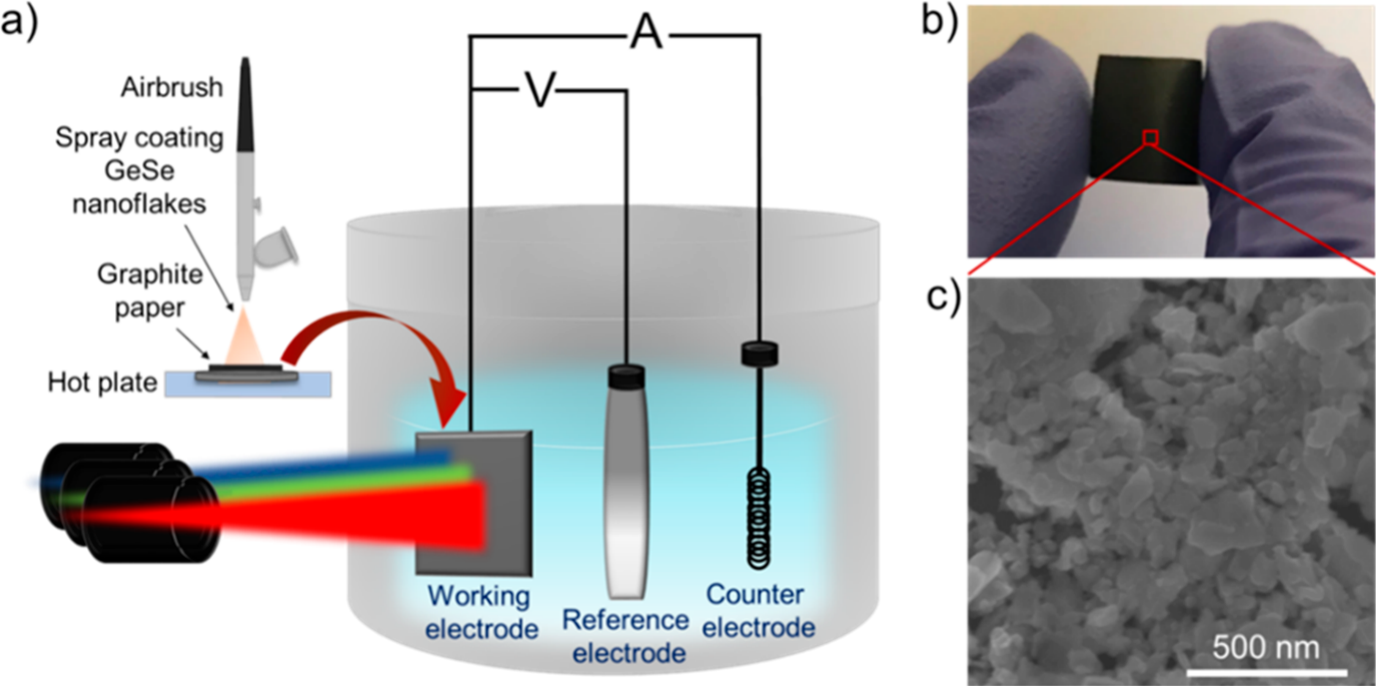
(a) Diagram of the experimental setup for electrochemical characterization of the GeSe photoelectrodes, which were produced by spray coating the GeSe nanoflakes onto a graphite paper substrate, acting as the current collector. (b) Photograph of a GeSe photoelectrode, which was manually bent to its mechanical flexibility, and (c) its corresponding top-view SEM image.

**Figure 6:**
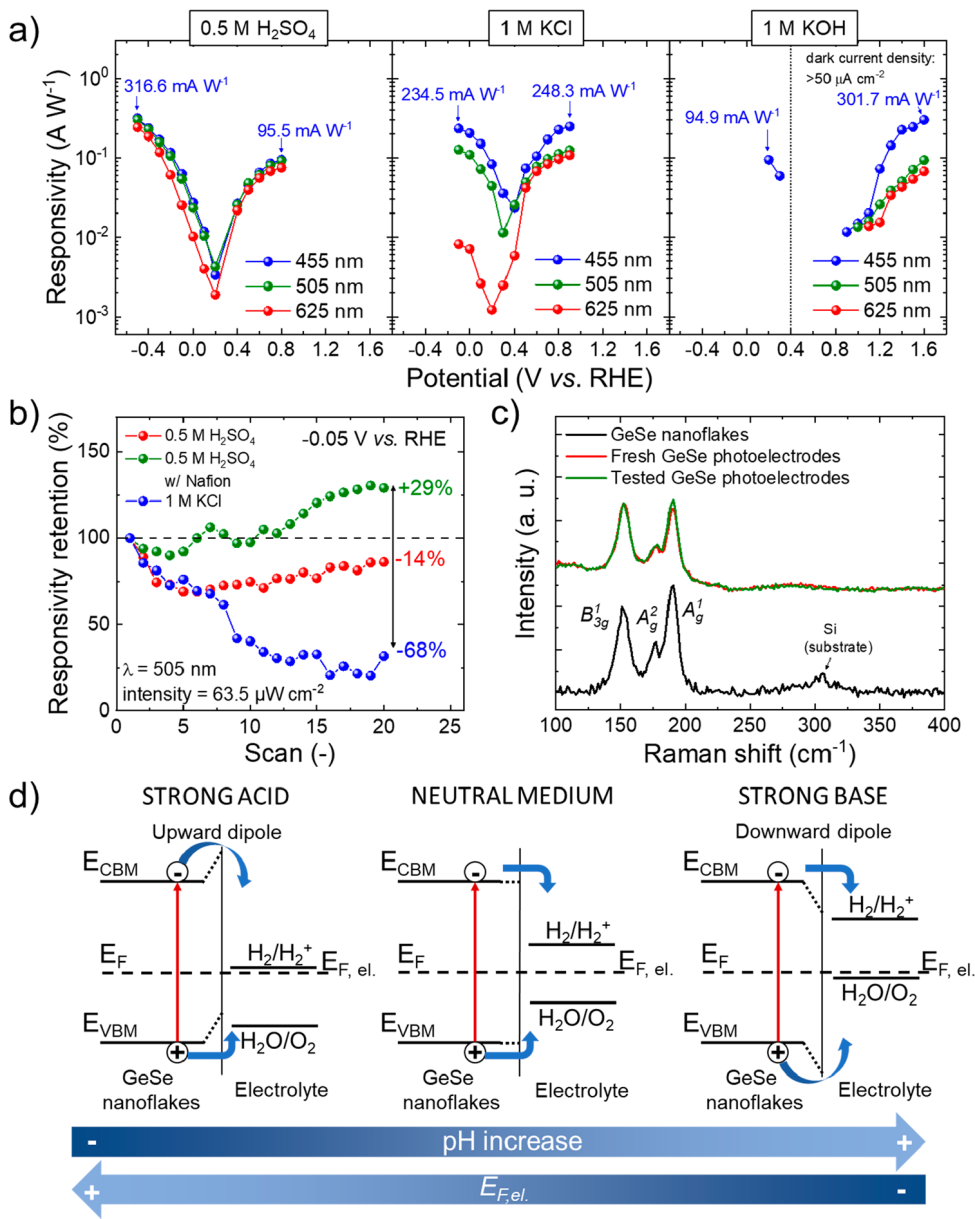
(a) Responsivity of PEC-type GeSe photodetectors as a function of the applied potential in the investigated media (e.g., 0.5 M H_2_SO_4_, 1 M KCl, 1 M KOH) upon three different illumination wavelengths in the visible spectral range: 455 nm, blue; 505 nm, green; and 625 nm, red. Light intensity: 63.5 μW cm^–2^. (b) Responsivity retention of the GeSe photodetectors in 0.5 M H_2_SO_4_ and 1 M KCl during cathodic operation (applied potential = −0.05 V vs RHE). (c) Raman spectra (*λ*_exc_ = 633 nm) of the GeSe nanoflakes deposited on Si substrate, fresh GeSe photoelectrodes, and tested Ge photoelectrodes (i.e., photoelectrodes measured after 20 cathodic LSV scans, as shown in panel b). (d) Sketch of the energy diagram at the GeSe nanoflake/electrolyte interfaces, pointing out the formation of upward and downward dipoles in strong acids and bases, respectively.

On the basis of our theoretical investigation of the photo(electro)catalytic properties of the single-/few-/multilayer GeSe flakes, the PEC properties of the LPE-produced GeSe nanoflakes were investigated for the water splitting reactions (HER and OER) in different aqueous media, ranging from acidic to alkaline solutions (i.e.: 0.5 M H_2_SO_4_, pH 0.3; 1 M KCl, pH 6.5; 1 M KOH, pH 14). The electrodes were produced by spray coating the GeSe nanoflake dispersion on graphite papers (mass loading of GeSe nanoflakes = 0.1 mg cm^–2^) and tested in a three-electrode configuration system (Figure 5a). To the best of our knowledge, a precise evaluation of the PEC properties of exfoliated GeSe materials in aqueous media is still missing, and only ref (68) has reported a preliminary PEC characterization of few-layer GeSe flakes in 0.1 M Na*_2_*SO_4_. Figure 5b reports a photograph of a sprayed GeSe photoelectrode, which was bent to evidence its mechanical flexibility. Figure 5c reports a top-view SEM image of a GeSe photoelectrode, showing a film made of nanoflakes preferentially placed with a horizontal position of their planes with respect to the substrate plane. The GeSe photoelectrodes were first evaluated as PEC-type photodetectors for three different illumination wavelengths in the visible spectral range, namely 455, 505, and 625 nm. These illumination wavelengths correspond to energies above the *E*_g_ of our GeSe nanoflakes, i.e., 1.27 eV (Figure 4c). Noteworthily, a photoresponse for these wavelengths can be used for the realization of colorimeters, i.e., three-channeled device that quantify the tristimulus red, green, and blue components by means photodetectors with spectral responsivity resembling the International Commission on Illumination (CIE)’s color matching functions (i.e., the numerical description of the chromatic responses of the CIE 1931 Standard Observer observer).^209−211^Figure 6a shows the responsivity vs potential plots derived from linear sweep voltammetry (LSV) measurements for GeSe photodetectors for illumination wavelengths (intensity = 63.5 μW cm^–2^) in 0.5 M H_2_SO_4_, 1 M KCl, and 1 M KOH. In order to avoid the photoelectrode degradation, the applied potentials were limited within a region corresponding to absolute dark current density inferior to 50 μA cm^–2^ for both cathodic and anodic operations (except for the anodic scans in 1 M KOH, in which higher dark current density were considered to display responsivities higher than 1 mA W^–1^). In all the investigated media, the responsivity of the photodetectors increases with decreasing the illumination wavelength. This behavior indicates that the photons with the highest energy (e.g., ∼2.7 eV for illumination wavelength = 455 nm) can efficiently excite the GeSe nanoflakes (in agreement with the DRS analysis, Figure 4c), including the thinnest ones, which exhibit the highest bandgap (1.80 eV for single-layer flakes, Figure 1b). For cathodic operation in 0.5 M H_2_SO_4_, the GeSe photodetectors reach a responsivity of 316.6 and 95.5 mA W^–1^ at −0.5 and +0.8 V vs RHE, respectively. In 1 M KCl, the photoelectrodes reach remarkable responsivity of 234.5 and 248.3 mA W^–1^ at −0.1 and +0.9 V vs RHE, respectively. The responsivity values of our GeSe photodetectors in both 0.5 M H_2_SO_4_ and 1 M KCl approach those of self-powered commercial UV–Vis photodetectors, including GaP or Si photodiodes.^212^ In addition, the responsivity is higher than the ones achieved by self-powered and low-voltage solution-processed photodetectors (see SI Table S2), including recent PEC-type photodetectors using group-IIIA metal monochalcogenides (e.g., InSe^60^ and GaSe^58^).

It is noteworthy that the highest recorded responsivity (i.e., 316.6 mA W^–1^) corresponds to an external quantum efficiency (EQE) (calculated as EQE = 100 × (responsivity/λ) × 1240 W nm A^–1^, in which λ is given in nm and the responsivity in A W^–1^) of 86.3%, thus approaching the theoretical performance limit for PEC-type photodetectors (i.e., 100%).^213^ The photoelectrodes in 1 M KOH display a cathodic responsivity of 94.9 mA W^–1^ at +0.2 V vs RHE. Notably, the graphite paper (substrates) shows significant (dark) current densities (on the order of tens of μA cm^–2^) during cathodic operation for potential inferior to +0.7 V vs RHE (Figure S6). These current densities restrict the analysis of the PEC properties of GeSe nanoflakes for potentials superior to +0.1 V vs RHE. Although the anodic responsivity of the GeSe photodetectors in KOH can reach values up to 301.7 mA W^–1^ at 1.6 V vs RHE, the high dark current density (>100 μA cm^–2^) suggests a possible corrosion of the photodetector materials. Since the graphite paper does not show significant current densities during anodic operation (Figure S7), the electrochemical reactivity of the photoelectrode materials can be mainly attributed to the GeSe nanoflakes. To preliminarily assess the stability of our photodetectors, their responsivity was evaluated over subsequent scans. During cathodic operation at −0.05 V vs RHE (Figure 6b), the photodetectors exhibited the most stable responsivity in 0.5 M H_2_SO_4_. In addition, a sulfonated tetrafluoroethylene-based fluoropolymer-copolymer (namely Nafion) film atop the photocatalytic GeSe film was evaluated to contrast a possible delamination of the GeSe nanoflakes as the redox reaction occurs.^214^ Consequently, the Nafion-coated photoelectrode did not exhibit any loss of responsivity, which, on the contrary, increased by +29%, possibly due to the hydration of the Nafion coating during subsequent LVS scans.^214,215^ The chemical and structural integrity of the GeSe nanoflakes after the stability test for cathodic operation in 0.5 M H_2_SO_4_ was further evaluated by Raman spectroscopy measurements (Figure 6c). The GeSe photoelectrodes show Raman spectra resembling the one measured on the as-produced GeSe photoelectrode and the GeSe nanoflakes (deposited onto a Si substrate). These results indicate that the GeSe nanoflakes retain their initial structural properties during cathodic operation. By increasing the pH, the photodetectors rapidly degrade, especially in 1 M KOH, in which they ceased to respond to light after two LSV scans. Figure S8 shows the chronoamperometry measurements performed on the GeSe photodetectors under 455 nm illumination at the fixed cathodic potential of −0.05 V vs RHE in the most stable electrolyte conditions, i.e., 0.5 M H_2_SO_4_ and 1 M KCl. In agreement with the LSV data, the GeSe photoelectrode retained its initial photocurrent in 0.5 M H_2_SO_4_, while a significant photocurrent degradation (∼−50%) was observed in 1 M KCl. The degradation of the photodetectors was also observed during anodic operation (Figure S9), although the highest responsivity retention was again observed in 0.5 M H_2_SO_4_. Although the theoretically predicted oxidation resistance of 2D GeSe,^68,132,133^ we cannot exclude that defective states in our nanoflakes can act as reactive sites triggering the oxidation processes. Prospectively, the production of undefective GeSe nanoflakes, through optimized synthesis protocols, may increase the stability and the reproducibility of the PEC performance of the GeSe nanoflakes.

Overall, our measurements revealed that GeSe nanoflakes can be used as a photocatalyst for water splitting reactions, but precautions are needed when selecting the electrolytic medium to avoid degradation effects. Our results suggest that acidic media and cathodic operation are adequate working conditions to stably exploit the photocatalytic properties of the GeSe nanoflakes. By deeply analyzing the potential dependence of the responsivity, we point out that the GeSe photodetectors show the best PEC performance at potentials relevant for water splitting applications (i.e., potential between 0 and +1.23 V vs RHE) in 1 M KCl. Since *E*(H^+^/H_2_) and *E*(O_2_/H_2_O) increase with increasing the pH, acidic and alkaline conditions are commonly expected to increase the water splitting activity for HER and OER, respectively. However, the band bending or the dipole formation occurring at semiconductor (photocatalyst)/electrolyte interface can also significantly affect the PEC performance of a photoelectrode. These effects depend on both the nature of the semiconductor (*n*- or *p*-type) and the pH of the aqueous media, as sketched by the energy diagrams of the GeSe nanoflake/electrolyte interface in Figure 6d. In particular, for strong acidic media (i.e., high H^+^ concentration) and insufficient p-doping of the photocatalysts, an upward band bending/dipole results in an energy barrier that the electrons have to overcome to carry out the HER.^216,217^ An equivalent consideration can be drawn for explaining the downward band bending/dipole in strong alkaline media, in which the OER-activity of the GeSe starts at higher overpotential in comparison to those observed in both quasi-neutral and acidic media. Since the UPS analysis revealed that our GeSe nanoflakes are slightly *n*-type materials, the aforementioned effects could explain the best photoresponse of the GeSe photoelectrodes at potentials between 0 and +1.23 V vs RHE in 1 M KCl. To validate this explanation, the light intensity dependence of the cathodic responsivity was evaluated in 0.5 M H_2_SO_4_ at fixed potential of 0 V vs RHE (Figure S10), under which conditions the photodetectors have shown a satisfactory stability. Typically, the relationship between the photocurrent density and the light intensity follows the power equation photocurrent density ∝ (light intensity)γ,^218,219^ in which γ is a factor determining the response of the photocurrent to light intensity. A unity value for γ indicates the absence of charge recombination and trapping processes, while nonunity γ suggests a complex process of charge generation, recombination, and trapping phenomena within the photoactive material.^218,219^ In 0.5 M H_2_SO_4_, the power law fit gives a γ of 0.56, indicating significant charge recombination of the photogenerated charges as originated by the presence of an interfacial dipole. Differently, in 1 M KCl the power law fits to the experimental data with γ equal to 0.83 (Figure S11). This value indicates a satisfactory utilization of the photogenerated charges to carry out the redox reaction. As expected for 2D materials, this effect can be attributed to the intrinsic maximization of the electrochemically accessible surface area,^28,58,220^ as well as to the nearly zero distance between the photogenerated charges and the catalytic surface area.^28,58,220^

**Figure 7:**
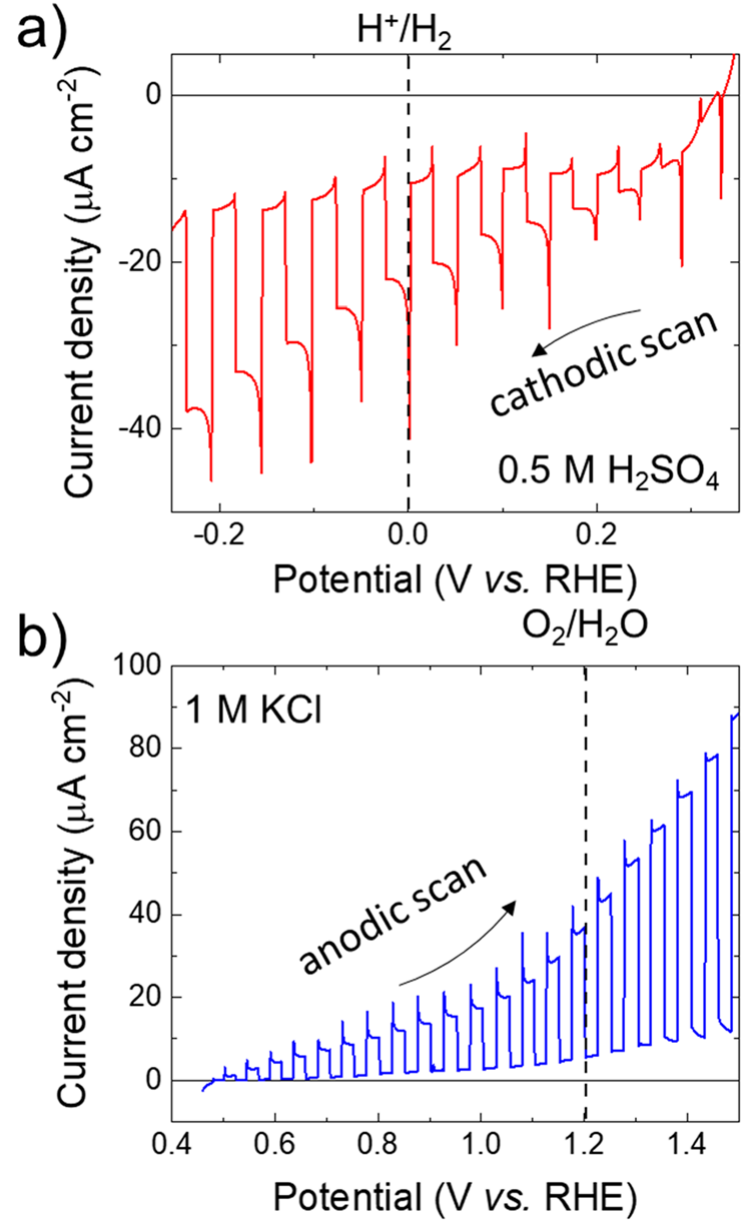
(a,b) LSV curves measured for GeSe photoelectrodes for (a) the HER (cathodic scan) in 0.5 M H_2_SO_4_ and (b) the OER (anodic scan) in 1 M KCl, under chopped simulated sunlight (i.e., AM 1.5G illumination). The redox potential for H^+^/H_2_ (0 V vs RHE) and O_2_/H_2_O (+1.23 V vs RHE) are also shown.

On the basis of the above PEC characterization, GeSe photoelectrodes were evaluated as either photocathodes or photoanodes for the HER and the OER, respectively, under chopped simulated sunlight (i.e., AM 1.5G standard spectra, irradiance = 1000 W m^–2^). Figure 7a,b show the cathodic and anodic LSV scans measured for GeSe photoelectrodes in 0.5 M H_2_SO_4_ and 1 M KCl, respectively. Noteworthy, 0.5 M H_2_SO_4_ and 1 M KCl media resulted in the highest photoelectrode photoresponses at 0 V vs RHE and +1.23 V vs RHE, respectively. The following Figures of Merit are used to quantify the photoresponse of the electrodes for water splitting reactions (i.e., HER and OER): the negative (cathodic) photocurrent density at 0 V vs RHE (*J*_0 V vs RHE_), the positive (anodic) photocurrent density at +1.23 V vs RHE (J_1.23 V vs RHE_) and the photocurrent onset potential (VOP) (defined as the equilibrium potential of the photoelectrodes under simulated sunlight). For the cathodic LSV scan in 0.5 M H_2_SO_4_, the GeSe photoelectrode shows: *J*_0 V vs RHE_ = −10.9 μA cm^–2^ and *V*_OP_ = +0.30 V vs RHE. For the anodic LSV scans in 1M KCl, the photoelectrode shows: *J*_1.23 V vs RHE_ = +31.0 μA cm^–2^ and *V*_OP_ = +0.48 V vs RHE. These results prove that the GeSe nanoflakes are promising materials to be used in water photoelectrolysis cells. Although it is beyond of the scope of the current work, our GeSe photoelectrodes could be further engineered by (1) adding charge selective layers to selectively control the interfacial charge transfer for a single photocarrier species (holes or electrons), making them either photocathodes or photoanodes; (2) incorporating cocatalysts onto their surface to further accelerate the water splitting reaction (e.g., 2D transition metal dichalcogenides for the HER^221−224^ and layered double hydroxide^225,226^ or functionalized graphene^227,228^ for the OER); (3) using porous substrates (e.g., carbon nanotubes),^229^ which mechanically stabilize photocatalytic materials on their surfaces; and (4) optimizing the thickness and the morphology of the sprayed GeSe film, thus increasing the overall absorption of the solar light.

## Conclusions

In summary, the electronic structure of GeSe has been theoretically studied using density functional theory (DFT) simulations. The calculated optical bandgap (*E*_g_) values (1.80 eV for single-layer flake and 1.35 eV for multilayer flakes) indicate that GeSe nanoflakes can optimally absorb the solar light. In addition, depending on the thickness and the pH, GeSe nanoflakes can fulfill one or both of the water splitting requirements, i.e., *E*_CBM_*> E*(H^+^/H_2_) and *E*_VBM_*< E*(O_2_/H_2_O). Our simulations show that GeSe nanoflakes with different thickness can be mixed in form of films to act as nanoscale tandem systems, in which the thinner nanoflakes operate as HER-photocatalysts, while the thicker ones perform the OER. Therefore, once coupled, GeSe nanoflakes in nanoporous films can intrinsically realize “all-solid-state Z-scheme water splitting pathways”, mimicking complex photosynthetic systems. On the basis of this theoretical prediction, a mixture of GeSe nanoflakes with different thicknesses, including single-/few-/multilayer flakes, has been produced through liquid-phase exfoliation (LPE) of a GeSe crystals in isopropyl alcohol (IPA). The GeSe photoelectrodes were produced by spray coating the as-produced GeSe nanoflake dispersion onto graphite papers, acting as the current collectors. The as-produced photoelectrodes were first investigated as photoelectrochemical (PEC)-type photodetectors in acidic (0.5 M H_2_SO_4_, pH 0.3), near neutral (1 M KCl, pH 6.5), and alkaline (1 M KOH, pH 14) media. In particular, the GeSe photodetectors reach responsivity up to 316.6 mA W^–1^ at −0.5 V vs RHE, which corresponds to an external quantum efficiency of 86.3%. This value approaches the theoretical limit of 100% of PEC-type photodetectors. Importantly, the GeSe photocathodes also stably operate. By increasing the pH toward alkaline values, the GeSe photodetectors start to degrade during operation, especially under anodic potential conditions. Lastly, the GeSe photoelectrodes were evaluated as photocathodes and photoanodes for HER and OER under simulated sunlight. The GeSe photocathodes reach a photocurrent density at 0 V vs RHE (*J*_0 V vs RHE_) of −10.9 μA cm^–2^ in 0.5 M H_2_SO_4_, while GeSe photoanodes display a photocurrent density at +1.23 V vs RHE (*J*_1.23 V vs RHE_) of 31.0 μA cm^–2^ in 1 M KCl. Overall, our evaluation of the photoelectrochemical (PEC) properties of GeSe nanoflakes in aqueous media can further spark the interest for novel type of water splitting photocatalysts based on group-IVA metal monochalcogenides. The engineering of GeSe photoelectrodes by optimizing the photocatalyst loading (i.e., film thickness), as well as by incorporating charge selective layers or cocatalysts, could prospectively boost the PEC performance of the GeSe nanoflakes achieved in this work. Nevertheless, the design of photoactive films composed by LPE-produced GeSe nanoflakes with different thicknesses, and thus different optoelectronic properties, could represent a straightforward approach to fabricate monolithic all-solid-state Z-scheme PEC devices.

## Supporting Information Available

Experimental section and additional TEM/SAED, SEM-EDS, XPS, DRS, and PEC characterizations (PDF)

## Abbreviations Used

PEC: photoelectrochemical
DFT: density functional theory
HER: hydrogen evolution reaction
OER: oxygen evolution reaction
CBM: conduction band minimum
VBM: and valence band maximum
IPA: isopropyl alcohol
LPE: liquid-phase exfoliation
