# Supplementary material for: Liquid-Phase Exfoliated GeSe Nanoflakes for Photoelectrochemical-Type Photodetectors and Photoelectrochemical Water Splitting
Source: ACS Appl Mater Interfaces. 2020 Sep 22;12(43):48598–613. doi: 10.1021/acsami.0c14201 (PMC8011798; doi:10.1021/acsami.0c14201)
Supplement: Supplementary file 1 — am0c14201_si_001.pdf [file am0c14201_si_001.pdf]

Supporting Information

Liquid-phase Exfoliated GeSe Nanoflakes for Photoelectrochemical-type Photodetectors and Photoelectrochemical Water Splitting

Gabriele Bianca,[†,⸸,‡] Marilena I. Zappia, [§,↧,‡] Sebastiano Bellani,[§,*] Zdeněk Sofer,[ǁ] Michele Serri,[†] Leyla Najafi,[§] Reinier Oropesa-Nuñez,[§,ǂ] Beatriz Martín-García,[†,#] Tomáš Hartman,[ǁ] Luca Leoncino,[˥] David Sedmidubský,[ǁ] Vittorio Pellegrini,[†,§] Gennaro Chiarello,[↧] and Francesco Bonaccorso[†,§,*]

[†]Graphene Labs, Istituto Italiano di Tecnologia, via Morego 30, 16163, Genova, Italy

[§]BeDimensional Spa., via Albisola 121, 16163 Genova, Italy

[⸸]Dipartimento di Chimica e Chimica Industriale, Università degli Studi di Genova, via Dodecaneso 31, 16146 Genoa, Italy

[ǁ]Department of Inorganic Chemistry, University of Chemistry and Technology Prague, Technická 5, 166 28 Prague 6, Czech Republic

[˥]Electron Microscopy Facility, Istituto Italiano di Tecnologia, via Morego 30, 16163 Genova, Italy

[↧]Department of Physics, University of Calabria, Via P. Bucci cubo 31/C 87036, Rende (CS), Italy

[ǂ]Department of Materials Science and Engineering, Uppsala University, Box 534, 75121 Uppsala, Sweden

[#]CIC nanoGUNE, 20018 Donostia-San Sebastian, Basque Country, Spain

[‡]These authors equally contributed

1. Experimental

Materials

Germanium (Ge) (99.9999 %) and selenium (Se) (99.999 %) were purchased from Strem Chemicals Inc. USA. Sulfuric acid (99.999%), KOH (≥ 85% purity, ACS reagent, pellets), KCl (ACS reagent, ≥ 99.0%) were purchased from Sigma Aldrich.

Theoretical calculations

Electronic structure calculations were performed using density functional theory (DFT) with generalized gradient approximation (GGA, PBE96 parametrization scheme)[1] and hybrid functionals (HSE06)[2], as implemented in MedeA-VASP software package,[3] for bulk and 1-, 2-, 4- and 6-layer (denoted as B and 1L, 2L,..,6L) GeSe by inserting ~20 Å-thick vacuum region between the respective slabs. Dispersion interactions within the simple VdW+D3-zero damping approximation[4] were considered in combination with GGA-PBE96 functional. The basis set was extended up to the cut-off energy 400 eV to increase the accuracy (a 300 eV cut-off was considered to calculate the non-local exchange interaction within HSE06). The k-point mesh was constructed inside the first Brillouin zone with k-point spacing smaller than 0.2 $Å^{-1}$. A tetrahedron integration scheme was applied for electron density of states calculation.

Synthesis and exfoliation of GeSe crystals

GeSe crystals were produced through direct synthesis (Bridgman-Stockbarger method) from powders of Ge and Se elements.[5] More in detail, an amount of Ge granules and Se powder (15 g) with an elemental stoichiometry of 1:1 were loaded in a quartz glass ampoule (25 mm × 150 mm), which was subsequently evacuated (pressure $<5 \times 10^{-3}$ Pa) using a diffusion pump. The evacuated ampoule was then sealed using an oxygen-hydrogen torch and then heated at 800 °C (*i.e.,* above melting point of GeSe) for 1 h (heating rate =

5 °C min^{-1}). The samples were then cooled down to room temperature (cooling rate = 0.3 °C min^{-1}), producing the GeSe crystal. The GeSe nanoflakes were produced by liquid-phase exfoliation (LPE)[6,7,8] in anhydrous 2-propanol (IPA) of the pulverized GeSe crystal, followed by sedimentation based separation (SBS)[7,9,10] to remove unexfoliated material. Experimentally, 50 mg of bulk crystals were added to 50 mL of anhydrous IPA and ultrasonicated in a bath sonicator (Branson® 5800 cleaner, Branson Ultrasonics) for 15 h. The resulting dispersions were ultracentrifuged at 700 g (Optima™ XE-90 with a SW32Ti rotor, Beckman Coulter) for 20 min at 15 °C in order to separate un-exfoliated bulk crystals (collected as sediment) from the exfoliated materials that remained in the supernatant. Then, the 80% of the supernatant was collected by pipetting, getting an exfoliated material dispersion.

Materials characterization

Scanning electron microscopy (SEM) analysis of the as-synthetized crystal was performed using a Tescan Lyra microscope at 15kV and 0.5 nA. The samples were imaged without any metal coating or pre-treatment. The EDS spectra were acquired with a microscope combined with an X-Max detector and INCA® system (Oxford Instruments), operating at 15kV and 0.8 nA. The samples were imaged without any metal coating or pre-treatment.

X-ray diffraction (XRD) measurements were acquired with a PANalytical Empyrean using Cu Kα radiation. The samples for XRD were prepared by depositing powder of GeSe crystal onto Si/SiO_2 substrates.

Transmission electron spectroscopy (TEM) images were acquired with a JEM 1011 (JEOL) TEM (thermionic W filament), operating at 100 kV. The morphological and statistical analyses were performed by using ImageJ software (NIH) and OriginPro 9.1 software (OriginLab), respectively. The samples for the TEM measurements were prepared by drop casting the as-prepared exfoliated material dispersions onto ultrathin C-on-holey C-coated Cu grids and rinsed with deionized water and subsequently dried overnight under vacuum. Selected Area Electron Diffraction (SAED) pattern was acquired with a JEM-1400Plus (JEOL) operating at 120kV equipped with LaB6 thermoionic source and with a CCD camera Orius 830 (Gatan). SAED pattern processing (azimuthal integration, background subtraction) was done using the PASAD plugin for Digital Micrograph.

The atomic force microscopy (AFM) images were acquired with a XE-100 AFM (Park System, Korea) by means of PPP-NCHR cantilevers (Nanosensors, Switzerland) having a tip diameter <10 nm. The images were collected in intermittent contact (tapping) mode on an area of 5×5 μm^2 (1024×1024 data points) using a drive frequency of ~330 kHz and keeping the working set point above 70% of the free oscillation amplitude. The scan rate for the acquisition of the images was 0.2 Hz. Gwyddion 2.54 software (http://gwyddion.net/) was used for processing the images and the height profiles, while the data were analyzed by using OriginPro 9.1 software. The latter was also used to carry out the statistical analysis on multiple AFM images (50 flakes) for all the tested samples. The samples were prepared by drop-casting the as-prepared GeSe nanoflake dispersion onto mica sheets (G250-1, Agar Scientific Ltd.) in N_2 and heating to 100°C for 15 min to dry the sample and remove adsorbates.

Raman spectroscopy measurements were performed by using a Renishaw microRaman Invia 1000 mounting a 50× objective, with an excitation wavelength of 633 nm and an incident power of 1 mW. For each sample, 50 spectra were collected. The samples were prepared by drop casting the as-prepared GeSe nanoflake dispersion onto Au-coated Si/SiO_2 substrates and subsequently dried under vacuum.

Diffusive reflectance spectroscopy (DRS) measurements were carried out on GeSe nanoflake dispersion (diluted or concentrated at various concentrations of GeSe nanoflakes) by using a Cary Varian 5000 UV−vis spectrometer with integrating sphere.

The X-ray photoelectron spectroscopy (XPS) analysis is accomplished on a Kratos Axis UltraDLD spectrometer at a vacuum < 10^{-8} mbar, using a monochromatic Al Kα source operating at 20 mA and 15 kV and collecting photoelectrons from a 300 × 700 μm^2 sample area. The charge compensation device was not used. Wide spectra were acquired at pass energy of 160 eV and energy step of 1 eV, while high-resolution spectra of Ge 2*p*, Ge 3*d*, Se 3*d*, O 1*s*, C 1*s* and Au 4*f* peaks were acquired at pass energy of 10 eV and energy step of 0.1 eV. The samples were prepared by drop-casting the dispersion of GeSe nanoflakes on an Au-coated Si substrate in N_2 atmosphere while heating the substrate to 120°C. As-synthetized GeSe crystals were stacked onto conductive carbon tape and cleaved prior analysis. The samples were then transferred

from air to the XPS chamber. Data analysis is carried out with CasaXPS software (version 2.3.19PR1.0). The energy scale was calibrated by setting the Au $4f^{7/2}$ peak at 84.0 eV. For a better comparison of the as-synthetized crystal and the GeSe nanoflakes, the spectra of the GeSe crystal were calibrated by setting the binding energy of the sharp Se 3*d* doublet equal to the one obtained in the nanoflakes.

Ultraviolet photoelectron spectroscopy (UPS) with He I (hν = 21.2 eV) radiation was performed to estimate the Fermi energy level (E_F) and the valence band maximum of the materials under investigation. The experiments were conducted on the samples after the XPS analysis using the same equipment. A −9.0 V bias was applied to the sample to precisely determine the low kinetic energy cut-off. The energy scale was corrected according to the binding energy calibration performed for the XPS measurement.

Electrodes fabrication

The photoelectrodes were produced by spray-coating the GeSe nanoflake dispersion (GeSe nanoflakes concentration = 0.13 g L^{-1}) onto graphite paper (PGS, Panasonic) mounted on a hot plate heated at 60 °C. The material mass loading was 0.1 mg cm^{-2}. The electrode area was 1.5×1 cm^2. The photoelectrodes were dried overnight at room temperature before the characterization.

Electrodes characterization

Scanning electron microscopy analysis of the of the as-produced electrodes was performed using a Helios Nanolab 600 DualBeam microscope (FEI Company) operating at 5 kV and 0.2 nA. The electrodes were imaged without any metal coating or pre-treatment.

The electrochemical measurements were performed at room temperature in a flat-bottom fused silica cell using the three-electrode configuration of the potentiostat/galvanostat station (VMP3, Biologic), controlled via own software. A glassy carbon rod and a KCl-saturated Ag/AgCl were used as the counter-electrode and the reference electrode, respectively. The measurements were carried out in 200 mL of 0.5 M H_2SO_4, or 1 M KCl, or 1 M KOH. The pH of the electrolytic solutions was measured with Oakton ION 700 ISE/pH Meter. Before starting the measurements, the oxygen was purged from electrolyte by flowing N_2 gas throughout the liquid volume using a porous frit. A constant, slight nitrogen flow is maintained afterwards for the whole duration of the experiments. The Nernst equation: $E_{RHE} = E_{Ag/AgCl} + 0.059 \times pH + E^0_{Ag/AgCl}$, where E_{RHE} is the converted potential *vs.* RHE, $E_{Ag/AgCl}$ is the experimental potential measured against the Ag/AgCl reference electrode, and $E^0_{Ag/AgCl}$ is the standard potential of Ag/AgCl at 25 °C (0.1976 V *vs.* RHE), was used to convert the potential difference between the working electrode and the Ag/AgCl reference electrode to the reversible hydrogen electrode (RHE) scale. The LSV curves were acquired at 5 mV s^{-1} scan rate, in both anodic and cathodic directions. The light emitting diodes (LEDs) M455L3 (Thorlabs), M505L3 (Thorlabs) and M625L3 (Thorlabs) were used as monochromatic source for λ of 455 nm, 505 nm and 625 nm, respectively. The light intensity of the LEDs was adjusted through source meter (2612B Dual-Channel System SourceMeter, Keithley)-controlled LED driver (LEDD1B, Thorlabs). The illumination intensity of the LED was calibrated by using an optical power and energy meter (PM100D, Thorlabs). A 300 W Xenon light source LS0306 (Lot Quantum Design), equipped with AM1.5G filters, was used to simulate solar illumination (1 sun). The responsivity of the photoelectrochemical (PEC)-type photodetectors was calculated by the ration between the photocurrent density and the light intensity at fixed wavelength. The main Figures of Merit (FoM) used to characterize the photocathodes for PEC water splitting are: the onset potential (V_{OP}), defined as the equilibrium potential of the photoelectrodes under illumination; the cathodic photocurrent density at 0 V *vs.* RHE (J_{0V} vs RHE) and the anodic photocurrent density at 1.23 V *vs.* RHE (J_{0V} vs RHE). The stability of the GeSe photodetectors was evaluated by recording subsequent 20 LSV scans and measuring the corresponding responsivity.

2. Selected-area electron diffraction analysis

Figure S1a shows the SAED pattern of the TEM image of the GeSe flakes. **Figure S1b** displays the background-subtracted and azimuthally integrated SAED pattern, which matches with the XRD pattern of the orthorhombic (*Pnma*) GeSe (ICSD card Nr. 230429) (**Figure S1c**).

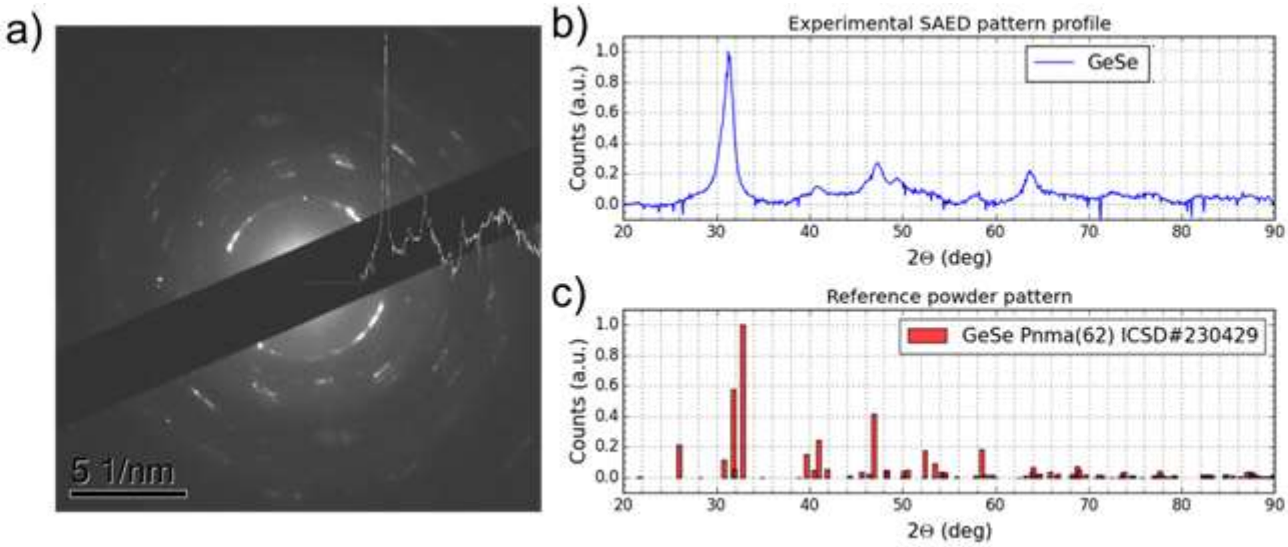

Figure S1. a) SAED pattern of the TEM image of the GeSe flakes. b) Background-subtracted and azimuthally integrated SAED pattern, plotted as a function of the Bragg angle calculated for Cu Kα wavelength c) XRD pattern of the orthorhombic (*Pnma*) GeSe (ICSD card Nr. 230429).

3. Raman statistical analysis

Figure S2 reports the statistical analysis of the B_{3g}, A_g^2 and A_g^1 Raman peaks for the GeSe bulk (panels a-c) and nanoflakes (panels d-f).

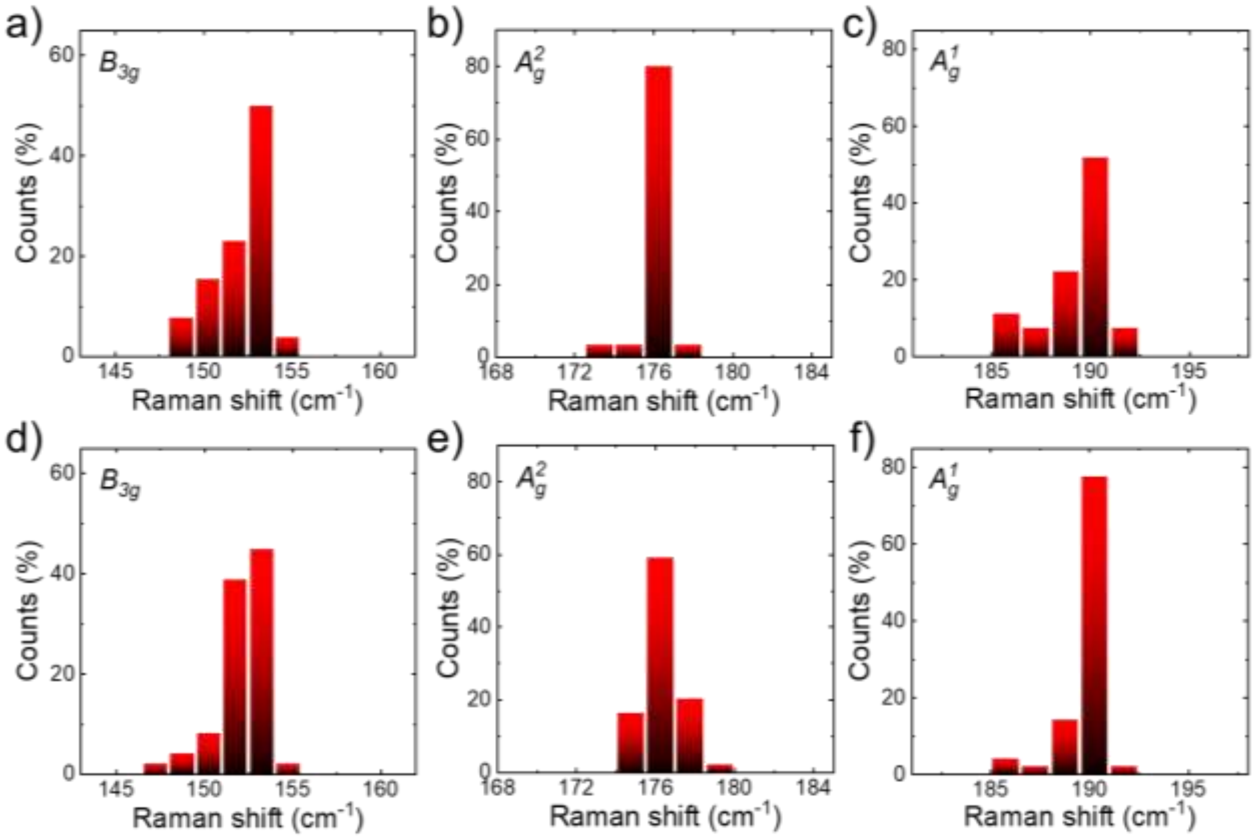

Figure S2. Statistical analysis of the B_{3g}, E_g^2 and E_g^1 Raman peaks for the a-c) GeSe bulk and d-f) nanoflakes.

4. Scanning electron microscopy-coupled energy dispersive X-ray spectroscopy analysis of the GeSe crystals

The chemical composition of the synthetized GeSe crystals was estimated through scanning electron microscopy (SEM)-coupled energy dispersive X-ray spectroscopy (EDS) analysis, whose images are reported in the main text (Figure 3b,c). As shown in **Table S1**, the SEM-EDS data indicate a slight Ge-enriched phases of the GeSe crystals (Ge-to-Se atomic ratio ~1.2). The excess of Ge compared to the ideal GeSe phase could be ascribed to the formation of surface oxides (*e.g.*, GeO_2), which protect the underlying GeSe from further oxidation.[11,5,12]

Table S1. Elemental composition of the GeSe crystals derived from the SEM-coupled EDS analysis.

**Element**	**atomic %**
Ge	55.0
Se	45.0

5. X-ray photoelectron spectroscopy analysis of the GeSe crystals and nanoflakes

The composition and the chemical state of the bulk and exfoliated GeSe were evaluated by XPS analysis (**Figures S3** and **S4**). For GeSe bulk, the high-resolution XPS spectrum of the Ge 3*d* region can be deconvoluted with multiple doublets, each one with a spin-orbit splitting of 0.6 eV.[13] The doublet with the highest intensity was attributed to the Ge(2+) of GeSe phase ($3d^{5/2}$ peak at binding energy – B.E. – of ~30.1eV).[14] The components at higher binding energy are attributed to Ge(4+) in Se-rich Ge selenides ($3d^{5/2}$ peak at ~30.6),[15] GeO ($3d^{5/2}$ peak at ~31.4 eV) and GeO_2 (unresolved doublet, 3*d* peak at ~32.9 eV)[16] present on the surface of the crystals. The high-resolution XPS spectrum of the Se 3*d* region can be fitted with two doublets corresponding to Se(2-) two-fold coordinated with Ge atoms as in GeSe ($3d^{5/2}$ peak at ~54.0 eV), and Se atoms with a fraction of homopolar Se-Se bonds ($3d^{5/2}$ peak at ~54.6 eV), that can be part of Se-rich Ge selenides. The area of the Ge 3*d* and Se 3*d* peaks, corrected by the relative sensitivity factors, gives a Ge/Se ratio of ~1.3, in agreement with the SEM-coupled EDS analysis of the GeSe crystals.

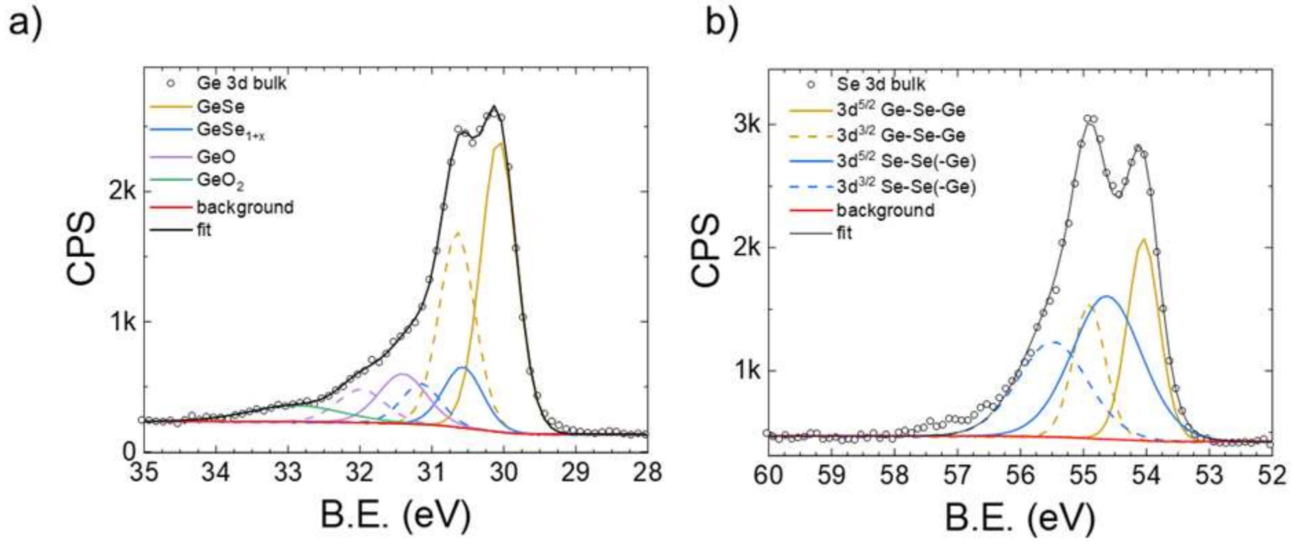

Figure S3. X-ray photoelectron spectroscopy analysis of the GeSe bulk. a) Ge 3*d* region with the deconvoluted components. b) Se 3*d* region with the deconvoluted components. Solid and dashed lines indicate the $3d^{5/2}$ and $3d^{3/2}$ components, respectively.

For the GeSe nanoflakes, the high-resolution XPS spectrum of the Ge 3*d* region can be deconvoluted with two doublets corresponding to GeSe ($3d^{5/2}$ peak at ~30.1eV) and to surface Ge(4+) selenides ($3d^{5/2}$ peak at ~30.7). Germanium oxides are not resolved in the exfoliated sample. The high-resolution XPS spectrum of the Se 3*d* region can be fitted with two doublets corresponding to Se belonging to GeSe ($3d^{5/2}$ peak at ~54.0 eV), and Se atoms with a fraction of homopolar Se-Se bonds ($3d^{5/2}$ peak at 54.5 eV) that are likely present on the surface of the flakes as a Se-rich Ge selenides. The area of the Ge 3*d* and Se 3*d* peaks, corrected by the relative sensitivity factors, gives a ratio between Ge and Se of ~1.0, as expected from the GeSe stoichiometry.

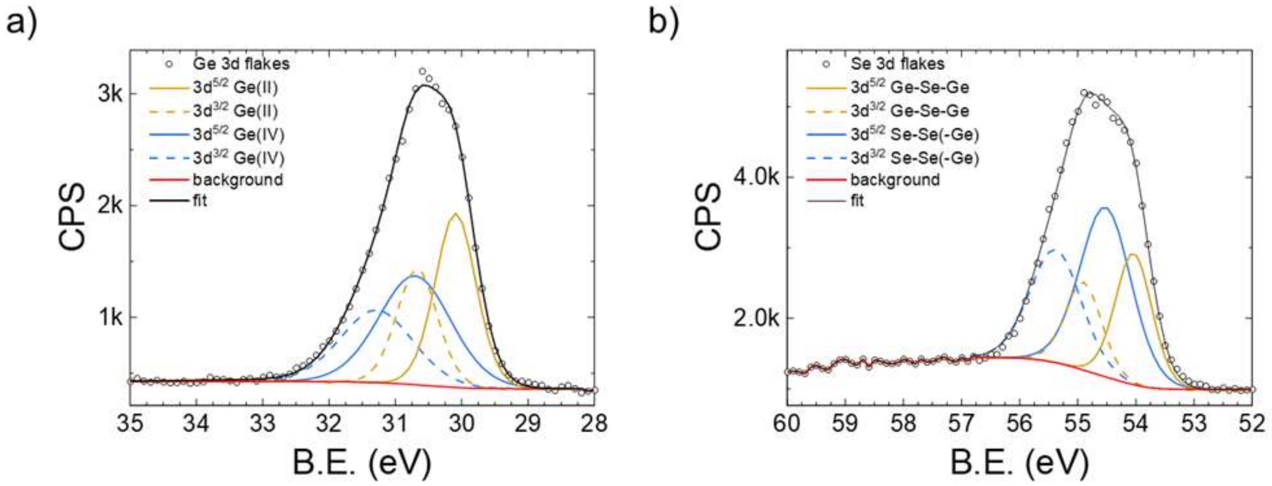

Figure S4. X-ray photoelectron spectroscopy analysis of the GeSe nanoflakes. a) Ge 3*d* region with the deconvoluted components. b) Se 3*d* region with the deconvoluted components. Solid and dashed lines indicate the $3d^{5/2}$ and $3d^{3/2}$ components, respectively.

6. Diffusive reflectance of a spray-coated film of GeSe nanoflakes

Figure S5 reports the *R* spectrum of a film of GeSe nanoflakes deposited on quartz substrate. These data have been used in the main text for the Tauc analysis of the GeSe nanoflakes.

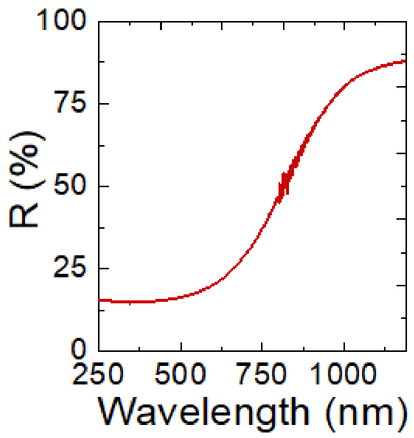

Figure S5. Reflectance spectrum (*R*) of a film of GeSe nanoflakes.

7. **Comparison between the responsivities of our PEC-type GeSe photodetectors and other solution-processed photodetectors reported in literature.**

Table S2. Comparison between the responsivities our PEC-type GeSe photodetectors and other solution-processed photodetectors reported in literature.

**Materials**	**Device configuration**	**Measurement conditions**		**Responsivity (mA W^{-1})**	**Illumination intensity (mW cm^{-2})**	**Wavelength (nm)**	**Reference**
		**Electrolyte**	**Applied potential**				
GeSe nanoflakes	PEC-type	0.5 M H_2SO_4	-0.5 V *vs.* Ag/AgCl	316.6	0.056	455	This work
		1 M KCl	-0.1 V *vs.* RHE	234.5	0.056	455	
GaSe nanoflakes	PEC-type	0.5 M H_2SO_4	-0.3 V *vs.* RHE	~160	< 0.0567	455	[17]
				19.5	31.8	455	
InSe nanosheets	PEC-type	0.2 M KOH	1 V *vs.* SCE	3.3×10^{-3}	120	Simulated sunlight	[18]
				4.9×10^{-3}	40	Simulated sunlight	
Black phosphorous nanosheets	PEC-type	0.1 M KOH	0 V *vs.* SCE	1.9×10^{-3}	20	Simulated sunlight	[19]
				2.2×10^{-3}	100	Simulated sunlight	
GeSe nanosheets	PEC-type	0.1 M KOH	0.3 V	0.044	118	Simulated sunlight	[20]
				0.076	26.2	Simulated sunlight	
SnS	PEC-type	0.1 Na_2SO_4	0.6 V	0.018	3.57	365	[21]
Perovskite ($CH_3NH_3PbI_3$)	Metal-semiconductor-metal	-	5 V	4.4	1	633	[22]
PBDTT-ffQx/PCBM bulk heterojunction	Metal-semiconductor-metal	-	10 V	1.15×10^{3}	25	365	[23]
SnS/RGO hybrid nanosheets	FET	-	V_{DS} = 5V, V_g = 0 V	180	0.12	Visible light	[24]
InSe nanoflakes	Metal-semiconduc	-	2 V	274000 A W^{-1}	0.53	455	[25]

	tor-metal						
Perovskite ($CH_3NH_3PbI_3$) PDPP3T	Metal-semiconductor-metal	-	1 V	10.7	0.5	365	[26]
				25.5		650	
				5.5		937	

8. Linear sweep voltammetry analysis of the graphite paper in 1 M KOH

Figure S6 shows the cathodic LSV curves of the graphite paper in 1 M KOH (*i.e.*, the current collector used for GeSe photoelectrodes). These data indicate that the graphite papers exhibit significant (dark) current densities (in the order of tens µA cm^{-2}) during cathodic operation for potential inferior to +0.7 V *vs.* RHE.

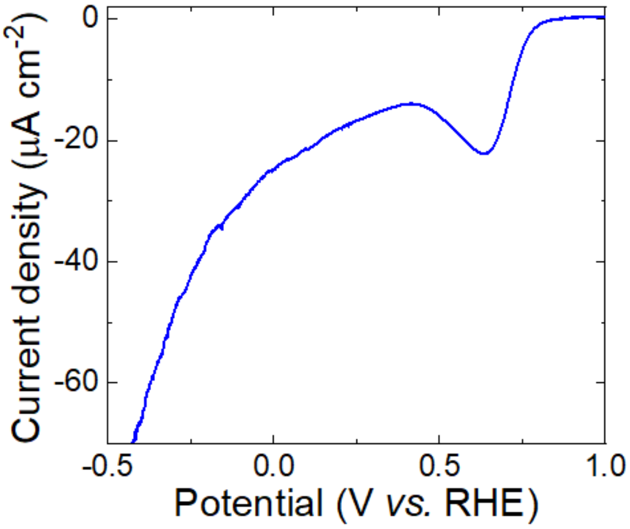

Figure S6. Cathodic LSV curve of the graphite paper in 1 M KOH.

Figure S7 reports the anodic LSV curves of the graphite paper in 1 M KOH. The current density is inferior to 10 µA cm^{-2}, indicating that the higher dark current observed for GeSe photoelectrodes for the same applied potential are attributed to GeSe nanoflakes.

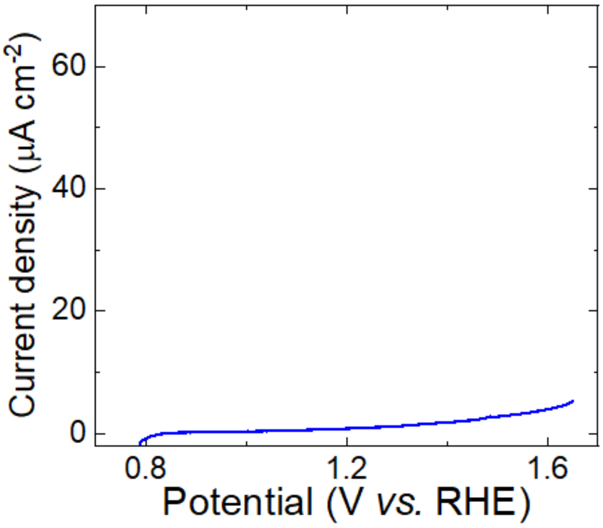

Figure S7. Anodic LSV curve of the graphite paper in 1 M KOH.

9. Stability tests of the GeSe photodetectors during anodic operation

Figure S8a,b show the chronoamperometry measurements performed on the GeSe photodetectors under 455 nm illumination (light intensity: 63.5 µW cm^{-2}) at -0.05 V *vs.* RHE, in the 0.5 M H_2SO_4 and 1 M KCl, respectively. After 3 min, the GeSe photodetector retained its initial photocurrent in 0.5 M H_2SO_4, supporting the stable behaviour observed during LSV analysis. In 1 M KCl, the photodetector progressively degraded, showing a ~50% photocurrent decrease after 3 min.

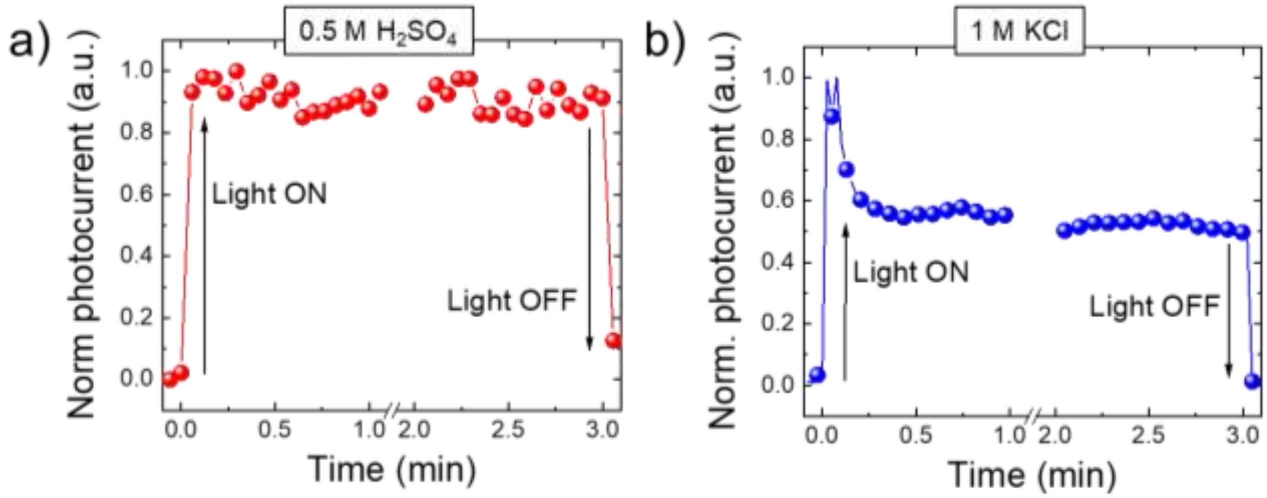

Figure S8. Choronoamperometry measurements for GeSe photoelectrode in a) 0.5 M H_2SO_4 and b) 1 M KCl under 455 nm illumination (light intensity: 63.5 µW cm^{-2}) at -0.05 V *vs.* RHE.

10. Stability tests of the GeSe photodetectors during anodic operation

Figure S9 reports the responsivity retention of the GeSe photodetectors over 20 anodic LSV scans in the investigated media, *i.e.*, 0. 5 M H_2SO_4, 1 M KCl and 1 M KOH. In all the case, the photodetectors significantly degrade over subsequent LSV scans, suggesting a possible oxidation of the GeSe flakes under anodic potentials.

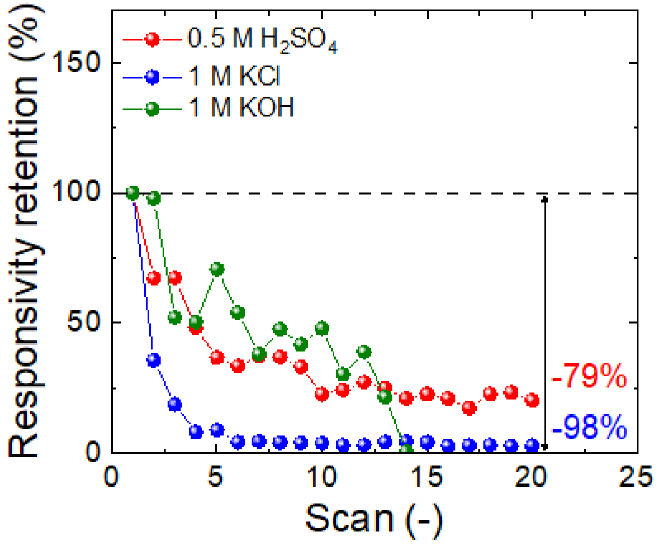

Figure S9. Responsivity retention of the GeSe photodetectors during anodic operation in 0.5 M H_2SO_4 (applied potential = +0.4 V *vs.* RHE), 1 M KCl (applied potential = +0.9 V *vs.* RHE) and 1 M KOH (applied potential = +1.2 V *vs.* RHE).

11. Light intensity dependence of the responsivity of the GeSe photodetectors

Typically, the relationship between the photocurrent density and the light intensity follows the power equation photocurrent density ∝ (light intensity)γ,[27,28] in which γ is a factor determining the response of the photocurrent to light intensity. A unity value for γ indicates the absence of charge recombination and trapping processes, while non-unity γ suggests a complex process of charge generation, recombination, and trapping phenomena within the photoactive material.[27,28] **Figure S10** shows the light intensity dependence of the (absolute) cathodic photocurrent density and the responsivity of the GeSe photodetectors in 0.5 M H_2SO_4 at fixed potential of 0 V *vs.* RHE, under which conditions the photodetectors showed a satisfactory stability. The power law fit of the photocurrent density gives a γ of 0.56, indicating significant charge recombination of the photogenerated charges, as expected when an interfacial dipole causes a barrier for the photogenerated charge to carry out the redox reaction. Therefore, the cathodic responsivity decreases with increasing the light intensity.

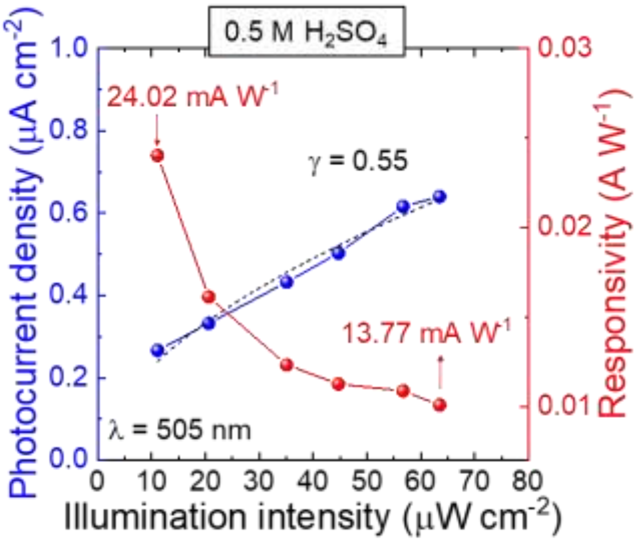

Figure S10. Absolute photocurrent density (left y-axis) and responsivity (right y-axis) of the GeSe photodetectors as a function of the light intensity measured at 0 V *vs.* RHE in 0.5 M H_2SO_4. The curves fitting the photocurrent density data is also shown (dashed black line).

In 1 M KCl, the power law fits to the experimental (absolute) photocurrent density values with γ equal to 0.83 (**Figure S11**). As discussed in the main text, this value indicates a satisfactory utilization of the photogenerated charges to carry out the redox reaction. As expected for 2D materials, this effect can be attributed to the intrinsic maximization of the electrochemically accessible surface area,[29,17,30] as well as to the nearly zero distance between the photogenerated charges and the catalytic surface area.[29,17,30] Consequently, the cathodic responsivity is well-retained with increasing the light intensity. Noteworthy, some losses of the cathodic photoresponsivity could be ascribed to the degradation of the GeSe photodetectors in 1 M KCl, as shown in the main text (Figure 6b).

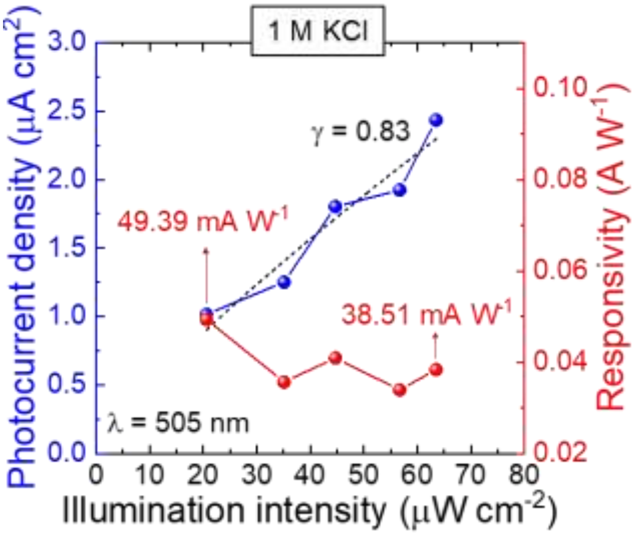

Figure S11. Absolute photocurrent density (left y-axis) and responsivity (right y-axis) of the GeSe photodetectors as a function of the light intensity measured at 0 V *vs.* RHE in 1 M KCl. The curves fitting the photocurrent density data is also shown (dashed black line).

References

(1) Perdew, J. P.; Burke, K.; Ernzerhof, M. Generalized Gradient Approximation Made Simple. *Phys. Rev. Lett.* **1996**, *77* (18), 3865–3868.

(2) Heyd, J.; Scuseria, G. E.; Ernzerhof, M. Hybrid Functionals Based on a Screened Coulomb Potential. *J. Chem. Phys.* **2003**, *118* (18), 8207–8215.

(3) Kresse, G.; Furthmüller, J. Efficient Iterative Schemes for Ab Initio Total-Energy Calculations Using a Plane-Wave Basis Set. *Phys. Rev. B - Condens. Matter Mater. Phys.* **1996**, *54* (16), 11169–11186.

(4) Grimme, S.; Antony, J.; Ehrlich, S.; Krieg, H. A Consistent and Accurate Ab Initio Parametrization of Density Functional Dispersion Correction (DFT-D) for the 94 Elements H-Pu. *J. Chem. Phys.* **2010**, *132* (15), 154104.

(5) Tan, S. M.; Chua, C. K.; Sedmidubský, D.; Sofer, Z. B.; Pumera, M. Electrochemistry of Layered GaSe and GeS: Applications to ORR, OER and HER. *Phys. Chem. Chem. Phys.* **2016**, *18* (3), 1699–1711.

(6) Bonaccorso, F.; Bartolotta, A.; Coleman, J. N.; Backes, C. 2D-Crystal-Based Functional Inks. *Adv. Mater.* **2016**, *28* (29), 6136–6166.

(7) Ciesielski, A.; Samorì, P. Grapheneviasonication Assisted Liquid-Phase Exfoliation. *Chem. Soc. Rev.* **2014**, *43* (1), 381–398.

(8) Nicolosi, V.; Chhowalla, M.; Kanatzidis, M. G.; Strano, M. S.; Coleman, J. N. Liquid Exfoliation of Layered Materials. *Science* **2013**, *340* (6139), 1226419–1226419.

(9) Khan, U.; O'Neill, A.; Porwal, H.; May, P.; Nawaz, K.; Coleman, J. N. Size Selection of Dispersed, Exfoliated Graphene Flakes by Controlled Centrifugation. *Carbon* **2012**, *50* (2), 470–475.

(10) Backes, C.; Abdelkader, A. M.; Alonso, C.; Andrieux-Ledier, A.; Arenal, R.; Azpeitia, J.; Balakrishnan, N.; Banszerus, L.; Barjon, J.; Bartali, R.; Bellani, S.; Berger, C.; Berger, R.; Ortega, M. M. B.; Bernard, C.; Beton, P. H.; Beyer, A.; Bianco, A.; Bøggild, P.; Bonaccorso, F.; Barin, G. B.; Botas, C.; Bueno, R. A.; Carriazo, D.; Castellanos-Gomez, A.; Christian, M.; Ciesielski, A.; Ciuk, T.; Cole, M. T.; Coleman, J.; Coletti, C.; Crema, L.; Cun, H.; Dasler, D.; De Fazio, D.; Díez, N.; Drieschner, S.; Duesberg, G. S.; Fasel, R.; Feng, X.; Fina, A.; Forti, S.; Galiotis, C.; Garberoglio, G.;

García, J. M.; Garrido, J. A.; Gibertini, M.; Gölzhäuser, A.; Gómez, J.; Greber, T.; Hauke, F.; Hemmi, A.; Hernandez-Rodriguez, I.; Hirsch, A.; Hodge, S. A.; Huttel, Y.; Jepsen, P. U.; Jimenez, I.; Kaiser, U.; Kaplas, T.; Kim, H. K.; Kis, A.; Papagelis, K.; Kostarelos, K.; Krajewska, A.; Lee, K.; Li, C.; Lipsanen, H.; Liscio, A.; Lohe, M. R.; Loiseau, A.; Lombardi, L.; López, M. F.; Martin, O.; Martín, C.; Martínez, L.; Martin-Gago, J. A.; Martínez, J. I.; Marzari, N.; Mayoral, Á.; McManus, J.; Melucci, M.; Méndez, J.; Merino, C.; Merino, P.; Meyer, A. P.; Miniussi, E.; Miseikis, V.; Mishra, N.; Morandi, V.; Munuera, C.; Muñoz, R.; Nolan, H.; Ortolani, L.; Ott, A. K.; Palacio, I.; Palermo, V.; Parthenios, J.; Pasternak, I.; Patane, A.; Prato, M.; Prevost, H.; Prudkovskiy, V.; Pugno, N.; Rojo, T.; Rossi, A.; Ruffieux, P.; Samorì, P.; Schué, L.; Setijadi, E.; Seyller, T.; Speranza, G.; Stampfer, C.; Stenger, I.; Strupinski, W.; Svirko, Y.; Taioli, S.; Teo, K. B. K.; Testi, M.; Tomarchio, F.; Tortello, M.; Treossi, E.; Turchanin, A.; Vazquez, E.; Villaro, E.; Whelan, P. R.; Xia, Z.; Yakimova, R.; Yang, S.; Yazdi, G. R.; Yim, C.; Yoon, D.; Zhang, X.; Zhuang, X.; Colombo, L.; Ferrari, A. C.; Garcia-Hernandez, M. Production and Processing of Graphene and Related Materials. *2D Mater.* **2020**, *7* (2), 22001.

(11) Rahaman, M.; Rodriguez, R. D.; Monecke, M.; Lopez-Rivera, S. A.; Zahn, D. R. T. GaSe Oxidation in Air: From Bulk to Monolayers. *Semicond. Sci. Technol.* **2017**, *32* (10), 105004.

(12) Chung, Y.; Lee, C.-W. Electrochemistry of Gallium. *J. Electrochem. Sci. Technol.* **2013**, *4* (1), 1–18.

(13) Goshtasbi Rad, M.; Göthelid, M.; Le Lay, G.; Karlsson, U. O.; Grehk, T. M.; Sandell, A. Influence of Charged Impurities on the Surface Phases of Sn/Ge(1 1 1). *Surf. Sci.* **2001**, *477* (2–3), 227–234.

(14) Mishra, P.; Lohani, H.; Kundu, A. K.; Patel, R.; Solanki, G. K.; Menon, K. S. R.; Sekhar, B. R. Electronic Structure of Germanium Selenide Investigated Using Ultra-Violet Photo-Electron Spectroscopy. *Semicond. Sci. Technol.* **2015**, *30* (7), 075001.

(15) Ciesielski, A.; Samorì, P. Graphene via Sonication Assisted Liquid-Phase Exfoliation. *Chemical Society Reviews*. 2014, pp 381–398.

(16) Oh, J.; Campbell, J. C. Thermal Desorption of Ge Native Oxides and the Loss of Ge Fom the Surface. *J. Electron. Mater.* **2004**, *33* (4), 364–367.

(17) Zappia, M. I.; Bianca, G.; Bellani, S.; Serri, M.; Najafi, L.; Oropesa-Nuñez, R.; Martín-García, B.; Bouša, D.; Sedmidubský, D.; Pellegrini, V.; Sofer, Z.; Cupolillo, A.; Bonaccorso, F. Solution-Processed GaSe Nanoflake-Based Films for Photoelectrochemical Water Splitting and Photoelectrochemical-Type Photodetectors. *Adv. Funct. Mater.* **2020**, *30* (10), 1909572.

(18) Li, Z.; Qiao, H.; Guo, Z.; Ren, X.; Huang, Z.; Qi, X.; Dhanabalan, S. C.; Ponraj, J. S.; Zhang, D.; Li, J.; Zhao, J.; Zhong, J.; Zhang, H. High-Performance Photo-Electrochemical Photodetector Based on Liquid-Exfoliated Few-Layered InSe Nanosheets with Enhanced Stability. *Adv. Funct. Mater.* **2018**, *28* (16), 1705237.

(19) Ren, X.; Li, Z.; Huang, Z.; Sang, D.; Qiao, H.; Qi, X.; Li, J.; Zhong, J.; Zhang, H. Environmentally Robust Black Phosphorus Nanosheets in Solution: Application for Self-Powered Photodetector. *Adv. Funct. Mater.* **2017**, *27* (18), 1606834.

(20) Ma, D.; Zhao, J.; Wang, R.; Xing, C.; Li, Z.; Huang, W.; Jiang, X.; Guo, Z.; Luo, Z.; Li, Y.; Li, J.; Luo, S.; Zhang, Y.; Zhang, H. Ultrathin GeSe Nanosheets: From Systematic Synthesis to Studies of Carrier Dynamics and Applications for a High-Performance UV–Vis Photodetector. *ACS Appl. Mater. Interfaces* **2019**, *11* (4), 4278–4287.

(21) Huang, W.; Xie, Z.; Fan, T.; Li, J.; Wang, Y.; Wu, L.; Ma, D.; Li, Z.; Ge, Y.; Huang, Z. N.; Dai, X.; Xiang, Y.; Li, J.; Zhu, X.; Zhang, H. Black-Phosphorus-Analogue Tin Monosulfide: An Emerging Optoelectronic Two-Dimensional Material for High-Performance Photodetection with Improved Stability under Ambient/Harsh Conditions. *J. Mater. Chem. C* **2018**, *6* (36), 9582–9593.

(22) Fang, H.; Li, J.; Ding, J.; Sun, Y.; Li, Q.; Sun, J. L.; Wang, L.; Yan, Q. An Origami Perovskite Photodetector with Spatial Recognition Ability. *ACS Appl. Mater. Interfaces* **2017**, *9* (12), 10921–10928.

(23) Tong, S.; Yuan, J.; Zhang, C.; Wang, C.; Liu, B.; Shen, J.; Xia, H.; Zou, Y.; Xie, H.; Sun, J.; Xiao, S.; He, J.; Gao, Y.; Yang, J. Large-Scale Roll-to-Roll Printed, Flexible and Stable Organic Bulk Heterojunction Photodetector. *npj Flex. Electron.* **2018**, *2* (1), 7.

(24) Zhuo, R.; Zuo, S.; Quan, W.; Yan, D.; Geng, B.; Wang, J.; Men, X. Large-Size and High Performance Visible-Light Photodetectors Based on Two-Dimensional Hybrid Materials SnS/RGO. *RSC Adv.* **2018**, *8* (2), 761–766.

(25) Curreli, N.; Serri, M.; Spirito, D.; Lago, E.; Petroni, E.; Martín-García, B.; Politano, A.; Gürbulak, B.; Duman, S.; Krahne, R.; Pellegrini, V.; Bonaccorso, F. Liquid Phase Exfoliated Indium Selenide Based Highly Sensitive Photodetectors. *Adv. Funct. Mater.* **2020**, *30* (13), 1908427.

(26) Chen, S.; Teng, C.; Zhang, M.; Li, Y.; Xie, D.; Shi, G. A Flexible UV–Vis–NIR Photodetector Based on a Perovskite/Conjugated-Polymer Composite. *Adv. Mater.* **2016**, *28* (28), 5969–5974.

(27) McGlynn, S. P. Concepts in Photoconductivity and Allied Problems. *J. Am. Chem. Soc.* **1964**, *86* (24), 5707–5707..

(28) Guo, F.; Yang, B.; Yuan, Y.; Xiao, Z.; Dong, Q.; Bi, Y.; Huang, J. A Nanocomposite Ultraviolet Photodetector Based on Interfacial Trap-Controlled Charge Injection. *Nat. Nanotechnol.* **2012**, *7* (12), 798–802.

(29) Su, T.; Shao, Q.; Qin, Z.; Guo, Z.; Wu, Z. Role of Interfaces in Two-Dimensional Photocatalyst for Water Splitting. *ACS Catal.* **2018**, *8* (3), 2253–2276.

(30) Wang, H.; Zhang, X.; Xie, Y. Recent Progress in Ultrathin Two-Dimensional Semiconductors for Photocatalysis. *Mater. Sci. Eng. R Reports* **2018**, *130*, 1–39.
